# A Synthetic Review of Cognitive Load in Distance Interpreting: Toward an Explanatory Model

**DOI:** 10.3389/fpsyg.2022.899718

**Published:** 2022-07-26

**Authors:** Xuelian Zhu, Vahid Aryadoust

**Affiliations:** ^1^Sichuan International Studies University, Chongqing, China; ^2^National Institute of Education, Nanyang Technological University, Singapore, Singapore

**Keywords:** cognitive load, causal factors, distance interpreting, measurement methods, pedagogical design

## Abstract

Distance Interpreting (DI) is a form of technology-mediated interpreting which has gained traction due to the high demand for multilingual conferences, live-streaming programs, and public service sectors. The current study synthesized the DI literature to build a framework that represents the construct and measurement of cognitive load in DI. Two major areas of research were identified, i.e., causal factors and methods of measuring cognitive load. A number of causal factors that can induce change in cognitive load in DI were identified and reviewed. These included factors derived from tasks (e.g., mode of presentation), environment (e.g., booth type), and interpreters (e.g., technology awareness). In addition, four methods for measuring cognitive load in DI were identified and surveyed: subjective methods, performance methods, analytical methods, and psycho-physiological methods. Together, the causal factors and measurement methods provide a multifarious approach to delineating and quantifying cognitive load in DI. This multidimensional framework can be applied as a tool for pedagogical design in interpreting programs at both the undergraduate and graduate levels. It can also provide implications for other fields of educational psychology and language learning and assessment.

## Introduction

Distance Interpreting (DI) refers to interpreting services provided by the interpreters who are geographically separate from clients and can only communicate through telephone calls or video links (Braun, 2020; AIIC, 2021). DI and onsite interpreting might share the same working mode, meaning that the interpreters listen and comprehend the source language and produce the target language either consecutively or simultaneously, but distance interpreters do so at different locations relative to the participants and with different technology requirements (Azarmina and Wallace, 2005; Braun and Taylor, 2012). The idea of distance interpreting meets with considerable support from the interpreting industry, represented by the AIIC’s endorsement. In its position document, AIIC states:

AIIC recognises that ICTs enable new interpreting modalities. These include setups whereby interpreters have no direct view of speakers/signers, but rather an indirect, ICT-enabled audio/audiovisual feed of speakers/signers who are not in the same physical location as interpreters, as well as setups where interpreters within the same team and even booth may be at different locations (AIIC, 2022).

Although the industry has recognized the position of interpreting in distance mode, studies have yet to comprehensively investigate this field. This is partly because distance interpreting was first applied in hospital and courtroom settings, in which consecutive mode is more widely applied; since DI requires constant turn taking to confirm information, the scope of research on DI is limited (Gracia-García, 2002; Jones et al., 2003; Braun, 2013). Another reason for the limitation of DI research might be a deficiency in technical support in the previous generation of DI, like limited bandwidth and video feed definition, which thoroughly hindered the wider spread of DI (Moser-Mercer, 2003; Ozolins, 2011). Currently, DI in simultaneous mode is commonly used with the support of technology and extended to live-streaming of videogames, sports narrations, and multilingual conferences that demand timely interpretation of different languages (Braun, 2020). These technologies have been particularly helpful during the COVID-19 pandemic, in which the need for DI services in both public service and conference settings was significantly heightened (Runcieman, 2020; Ait Ammour, 2021).

One of the recognized issues in DI is the role of cognitive load in performance (Moser-Mercer, 2005a,b; Mouzourakis, 2006), whose underlying mechanisms and effects have been examined in the published literature. Notably, Moser-Mercer (2005a) mentioned that fatigue during distance interpreting may be a “consequence of allocating additional cognitive resources” (p. 1). In line with this postulation, later studies investigated visual ecologies in DI to understand how and what visuals should be presented to interpreters during DI, as the visuals are additional input that may ensue change in cognitive load (Mouzourakis, 2006; Licoppe and Veyrier, 2017; Plevoets and Defrancq, 2018). In addition, Wessling and Shaw (2014) pointed out that the emotional state of distance interpreters, partly occasioned by cognitive load, might influence “longevity” in the field. However, these findings are tentative and inconclusive. Notably, there is no general framework to theorize and measure the extent to which different factors instigate change in cognitive load of DI interpreters. Understanding these factors, particularly the mode-specific nature of DI, is key to understanding whether the distance mode of working has a positive or adverse effect on the cognitive load of interpreters. To bridge this gap in knowledge, we review the causal factors inducing change in cognitive load in DI and methods of quantifying and measuring change in cognitive load.

In what follows, we will present a brief review of the scope of cognitive load theory, identify the causal factors inducing change in distant interpreters’ cognitive load, align these factors with pertinent measurement methods, and finally discuss the implications of the study for research. It should be noted that the study is focused on spoken language interpreting and excludes sign language interpreting.

## Cognitive Load Theory and Interpreting

Cognitive load (also known as mental workload, mental load, or mental effort) has drawn research interest from scholars in diverse disciplines (Sweller et al., 2011; Ayres et al., 2021; see also chapters in Zheng, 2018). Cognitive load is defined as the mental workload imposed on a performer when executing a particular task (Yin et al., 2008; Sweller et al., 2011; Sweller, 2018). Numerous studies have attempted to determine the constituents of cognitive load and how its components interact with each other (e.g., Moray, 1967; Hart and Staveland, 1988; Meshkati, 1983; unpublished Doctoral dissertation^1^, 1988; Young and Stanton, 2005; Pretorius and Cilliers, 2007; Byrne, 2013; Young et al., 2015; Kalyuga and Plass, 2018; Schnaubert and Schneider, 2022). Generally, these studies consider cognitive load as a multidimensional construct comprising several fundamental aspects, like tasks, operators, and context (Martin, 2018; Paas and van Merriënboer, 2020). Among them, cognitive load theory (CLT; Sweller et al., 2011) has offered important insights on the role of working memory, types of cognitive load, and the role of individual characteristics in cognitive tasks. In this theory, cognitive load consists of the *mental load* engendered by the task and environment factors and the *mental effort* or the cognitive resources allocated by the task performer to deal with task demands (Meshkati, 1988; Paas and Van Merriënboer, 1994; Sweller et al., 1998, 2019; Yin et al., 2008).

CLT was first introduced in the field of learning and instruction in the 1980s. The theory posits that new information (perceived stimulus) is first processed by working memory (WM) and then stored in long-term memory for future use (Sweller et al., 2011). In addition, in CLT, WM is postulated to have limited capacity, as visual and auditory channels compete for resources, while long-term memory is arguably limitless (Sweller, 1988). Due to the limited capacity of WM, it is crucial to maintain cognitive load at a manageable level to sustain productivity. Since WM is integral to the process of interpreting, it has been extensively discussed in the interpreting literature by many scholars (e.g., Moser-Mercer et al., 2000; Christoffels and De Groot, 2009; Seeber, 2011, 2013; Chmiel, 2018; Dong et al., 2018; Mellinger and Hanson, 2019; Wen and Dong, 2019; Bae and Jeong, 2021).

According to Sweller et al.’s (2011) tripartite model, there are three types of cognitive load: intrinsic, extraneous, and germane. Intrinsic cognitive load refers to the inherent difficulty of the information and the interactivity of the characteristics of the input; accordingly, task complexity depends on the nature and content of the information and the skills of the person who processes the information (Leppink et al., 2013). Extraneous cognitive load, on the other hand, is generated by the manner in which information is presented and whatever the learner (processor) is required to do and as such, it is under the control of task designers (Cierniak et al., 2009). In addition, germane cognitive load is required for learning, processing and (re)constructing information; it can compete with and occupy the WM resources that help with processing the intrinsic cognitive load (Paas and van Merriënboer, 2020). In instruction designs, it is recommended to limit extraneous cognitive load while promoting germane load so as to direct the learner’s attention to the cognitive processes that are relevant to the processing of key information (van Merriënboer et al., 2006). The same limit to extraneous cognitive load applies in interpreting. For example, a better booth design that blocks out environmental noise would provide a better venue for interpreters as it lowers the extraneous cognitive load caused by noise and allows interpreters to allocate their cognitive resources to the processing of intrinsic and germane cognitive loads.

Early models of cognitive load in interpreting studies were mainly drawn from conceptual discussions and, thus were backed up by little supporting data (Setton, 1999). In a novel theory for its time, Gerver (1975) argued that information is processed during simultaneous interpreting (SI) through “a buffer storage” (p. 127), which is separate for the source and target languages. Although there is yet no empirical support for this hypothesis, this view of storage aligns with the idea that informational sources can be processed in parallel (Timarová, 2008). Successively, Moser (1978) developed a process model of interpreting that placed generated abstract memory (GAM) at the center of discussion. She proposed that GAM is the equivalent of short-term memory, which was later reconceptualized as WM by Baddeley and Hitch (1974) and Baddeley (1986, 2000, 2012). Kirchhoff (1976) described cases in which the completion of tasks requires more processing capacity than is available to the interpreter.

In a similar vein, Gile’s (1995, 2009) effort model, Gile’s (1999) tightrope hypothesis, and Seeber’s (2011) cognitive load model all underscored the role of various factors in successful SI and had a strong impact on the interpreting research (Pöchhacker, 2016). Gile’s (1995, 2009) effort model is an operation model based on the theoretical assumption of limited attentional resources. The model assimilates interpreters to a tightrope walker who has to utilize nearly all their mental effort during interpreting, which is available only in limited supply. The interpreting process comprises three core efforts: comprehension, production and short-term memory. Comprehension is the process of perceiving and understanding the input, while production involves the articulation of the translated code. Short-term memory (STM) is an interpreter’s capacity to tentatively store limited bits of information. STM capacity is indicated, among other things, by ear-voice-span (EVS) during interpreting, which refers to the time lag between comprehension and production during which interpreters make decisions about their interpreting (Christoffels and De Groot, 2004; Chen, 2018; Collard and Defrancq, 2019; Seeber et al., 2020). Gile’s effort model provides a reliable representation of interpreting which may be useful in practice and pedagogical design in interpreting training programs. However, Seeber (2011) argues that Gile’s effort model, which is based on Kahneman’s (1973) single resource theory, assumes that interpreters draw resources from undifferentiated pools, so it is unable to identify interferences of subtasks. Built on this argument, Seeber’s (2011) cognitive load model is founded upon multiple resource theory (Wickens, 1984, 2002); it recognized and accounts for the conflict and overlap between language comprehension and production during interpreting. Seeber (2011) used this analytical framework to illustrate how the overall demand of interpreting is affected by comprehension and production tasks and their interference with each other. This approach to understanding cognitive load in interpreting broadens the scope of research on cognitive load and sets the stage for the integration of the multimodality approach in interpreting research (Seeber, 2017; Chmiel et al., 2020).

In our discussion, the cognitive load in distance interpreting as well as the general and interpreting-specific cognitive models will remain crucial to identifying the causal factors and their measurement methods.

## Overview of the Theoretical Framework of the Study

**Figure 1:**
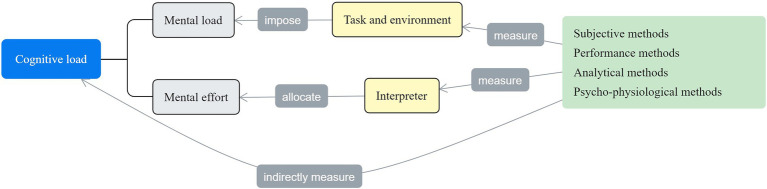
Representation of cognitive load and its measurement in DI.

As demonstrated in Figure 1, cognitive load in DI comprises mental load, mental effort, and pertinent measurement models (in the box on the right-hand side). Inspired by the CLT surveyed earlier, mental load refers to the cognitive demand of the DI tasks and environment, whereas mental effort refers to the total work done by the DI interpreter to complete the task. That is, task and environment factors impose mental load on the interpreter, who then devotes measurable mental efforts to perform the task (Meshkati, 1988; Yin et al., 2008; Chen, 2017).

Based on Paas et al. (2003) and Schultheis and Jameson (2004), the preceding factors in DI can be measured using four methods: subjective methods (Ivanova, 2000), performance methods (Han, 2015), analytical methods (Gile, 2009; Seeber, 2013) and psycho-physiological methods (Seeber and Kerzel, 2011). Subjective methods demand that participants rate the cognitive load they have experienced or are experiencing in a task; performance methods are used to measure cognitive load based on participants’ overt performance and behavior in DI; analytical methods are also subjective and based on the prior knowledge of the investigator who estimates or predicts the cognitive load of the input; and psycho-physiological methods are used to evaluate the neurophysiological processes underlying DI to infer the cognitive load that participants experience (Paas et al., 2003; Schultheis and Jameson, 2004).

As discussed later in this paper, the use of measurement methods in quantifying cognitive load in DI offers several advantages. First, they allow the researcher to quantify and measure cognitive load in DI as a multidimensional mental and verbal activity. The causal factors, which regulate change in cognitive load in DI, have the role of surrogates or indicators of immediate or cumulative cognitive load, so measuring these factors would provide an estimation of cognitive load in DI. Second, the measurement methods can be used to predict the mental effort that the distance interpreter may encounter by either subjective reports from the interpreter or objective measurement or analysis of the interpreter’s performance and/or their psycho-physiological reactions. As a result, stakeholders (such as conference organizers) might use measurement results to create more ergonomically effective working environments, thus allowing the interpreter to direct their mental effort to improving their performance. Third, the DI interpreting trainers use these measurement methods to estimate the mental load that task and environment factors impose on the trainees. This way, interpreter trainers can select proper training materials and design proper training curriculum to prepare the future DI interpreters.

We will further discuss the advantages (and shortcomings) of each measurement method in the context of DI, and how they may be used to quantify the indicators of cognitive load in DI.

## Causal Factors

### A General View of the Causal Factors

The cognitive capacity of DI interpreters is key to performing interpreting tasks. Cognitive capacity can be proxied by measurements of cognitive load consisting of mental load and mental effort. Understanding the task factors that induce change in mental load can help trainers to prepare the DI interpreters for complex interpreting tasks. Similarly, it is helpful to understand and measure interpreters’ mental effort to discriminate interpreter levels such as novice versus master interpreters, which, in turn, can provide diagnostic information in training programs.

However, cognitive load is a latent construct, which cannot be directly measured. Accordingly, measurement of cognitive load is carried out through delineating and operationalizing observable surrogates (indicators) that proxy cognitive load (Chen, 2017). In our study, the surrogates are called causal factors of cognitive load, meaning that these factors can *cause* change in cognitive load in DI—i.e., they have a cause-effect relationship with cognitive load. In theory, in a well-designed study where extraneous factors are properly controlled, the amount of cognitive load of DI interpreters can be directly manipulated by changing the magnitude of causal factors. A recent study by Braun (2020) shows that the causal factors in DI are more diverse than those in the related fields such as SI (e.g., Kalina, 2002; Campbell and Hale, 2003).

**Figure 2:**
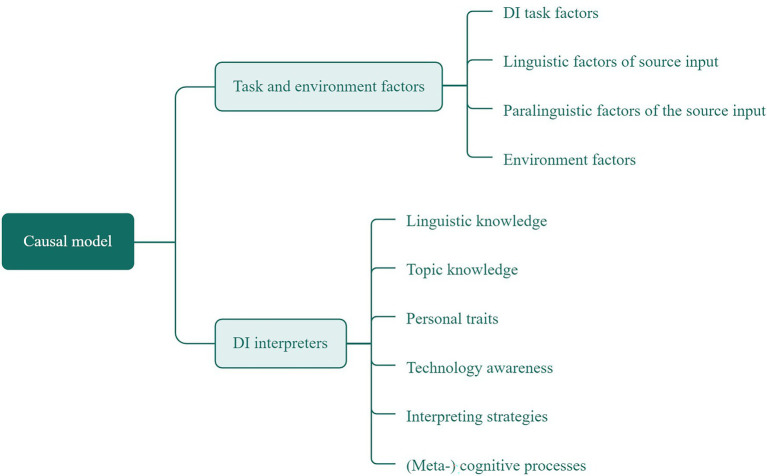
Causal factors that induce change in interpreters’ cognitive load during DI.

In the absence of a validated model for the factors that cause cognitive load in DI, we synthesized the extant literature in interpreting studies (e.g., Kalina, 2002; AIIC, 2021) and the communicative language ability framework (Bachman and Palmer, 1996), to present an integrative framework of DI that specifies causal factors (as demonstrated in Figure 2). The proposed model for causal factors in interpreting consists of two dimensions: (1) task and environment factors, and (2) interpreter factors. The task and environment factors comprise DI task factors, linguistic and paralinguistic features of the input, as well as the environment factors. The DI interpreter factors, on the other hand, comprise linguistic knowledge, topic knowledge, personal traits, technology awareness, interpreting strategies and (meta-)cognitive processes of the interpreters. It is suggested that the completion of an interpreting task is the result of the interaction between task and environment and interpreter characteristics. Even though these characteristics are defined separately, they are related, since there is no interpreting without an interpreter and there is no interpreter if there is no interpreting task in a given environment. Nevertheless, in experimental designs, investigators usually manipulate one or a few factors and keep the other factors constant to examine the change of cognitive load ensued from the manipulated factors (Choi et al., 2014). For interpreters with similar proficiency levels, better performance can be viewed as an indication of a lower cognitive load due to facilitating environments (e.g., interpreting from a home studio would be more convenient than interpreting from a booth at a conference room.). In experimental designs to examine cognitive load in DI, the causal factors can be perceived as independent variables which exert an influence over cognitive load, thus resulting in the difference in measurement and analysis results (e.g., interpreters’ performance quality and/or gaze behavior measured by eye-fixation indices).

The factors and sample studies in interpreting research are presented in Table 1. DI shares many features with onsite interpreting but differs from it in that the DI process is mediated by technology (Mouzourakis, 2006; Braun, 2020). These characteristics of DI add “another layer of complexity to communication” (Davitti and Braun, 2020, p. 281) and have the potential to cause significant change in interpreters’ cognitive load. For this reason, we italicized the factors that can significantly influence the DI mode to differentiate their mechanism from interpreting. Therefore, the italicized factors distinguish DI from onsite interpreting, while the non-italicized features are shared between the two. It should further be noted that the factors presented in this framework do not make an exhaustive and complete list but rather present a framework in-progress that is to be extended as further empirical and conceptual studies are conducted. From this perspective, the framework is reminiscent of the notion that “no model is meant to correspond exactly to the phenomena” (Moser-Mercer, 1997, p. 159) and as our knowledge about DI expands, so does the model. We will provide a detailed discussion of the causal factors and show how they can result in change in the cognitive load and performance of interpreters.

**Table 1:** An overview of previous studies on the causal factors that change interpreters’ cognitive load during interpreting.

General dimensions		Factors	Examples and/or explanation	Sample relevant studies
Task and environment factors	DI task factors	*Mode of presentation*	Audio only, video; Images: none, slides, online platform window (e.g., Zoom), stage view, head view, upper body view, spatial arrangements	Mouzourakis (2006); Braun and Taylor (2012)
		Materials and information specificity	Slides, speech draft, charts, reference list, website links	Kalina (2002); Díaz-Galaz et al. (2015)
		Turn duration	20 min per turn/ one speaker per turn	AIIC (2021)
		*Participant interactivity*	With an onsite audience, no onsite audience, platform audience (e.g., Zoom)	Napier et al. (2018)
		Directionality	L1-L2, L2-L1	Chen (2020)
	Linguistic factors of the source input	Lexical	Content word density, vocabulary difficulty	Korpal and Stachowiak-Szymczak (2018); Plevoets and Defrancq (2018)
		Syntactic	Simple sentences, compound sentences	Seeber and Kerzel (2011); Ma et al. (2021)
		Semantic	Culturally loaded expressions	Zheng and Zhou (2018)
		Textual organization	Cohesion, genre	Vianna (2005); Kuang and Zheng (2022)
		Pragmatic	Intention, implication	Setton (2002)
		Sociolinguistic	Language variety, register	Jenkins (2000)
	Paralinguistic factors of the source input	Delivery speed	Fast, normal, slow	Korpal and Stachowiak-Szymczak (2019)
		Accent	American English, Singlish	Gluszek and Dovidio (2010)
		*Body language*	Audio only (no body gestures at all) or video (with/without facial expressions, with/without upper body gestures)	Braun (2020); Vranjes and Oben (2022)
	Environment factors	*Booth type*	DSI hub, home studio, onsite booth, virtual booth	AIIC (2020a)
		*Platform*	Open or closed platforms	AIIC (2020b)
		Physical parameters	Acoustic condition, ventilation, lighting, air quality	Moser-Mercer (2003); Roziner and Shlesinger (2010)
		*Transmission techniques*	Onsite infrared/radio signals, ethernet, WiFi, mobile data; bandwidth	Mouzourakis (2006)
Interpreter’s factors		Linguistic knowledge	Knowledge of the language DI interpreters work in: grammatical knowledge, textual knowledge, functional knowledge, and sociolinguistic knowledge	Kalina (2000); Napier (2002); Li (2018)
		Topic knowledge	The content knowledge of the cultures the interpreters work in	Kalina (2000)
		Interpreting strategies	The skills to comprehend and produce languages	Chang and Schallert (2007); Ma and Li (2021); Amos et al. (2022)
		Personal traits	Personal factors like motivation, anxiety and stress resistance	Chiang (2009); Rojo López et al. (2021); Kuang and Zheng (2022)
		Technology awareness	Being mindful of the use of technology and the ability to use technology in interpreting	Desmet et al. (2018); Prandi (2018)
		(meta-)cognitive processes	The ability to build up a mental representation; meta-cognitive processes to plan, monitor and evaluate	Cañada and Arumí (2012); Lin et al. (2018); Bravo (2019)

### Task and Environment Factors

In a cognitive load model, task and environment factors include task criticality and psychological and environmental factors (Meshkati, 1988). There are four general factors that constitute the task and environment factors, namely DI task factors, linguistic factors of source input, paralinguistic factors of source input and environment factors. They are discussed below.

*DI task factors* are the mode of presentation, materials received, information specificity, DSI turn duration, participant interactivity, and directionality. With respect to the mode of presentation, DI interpreters may receive audiovisual input, audio only, or video (Braun, 2015). It remains inconclusive as to whether the use of one modality (audio only) or two modalities (audio and visual input) can reduce the cognitive load of DI interpreters (Moser-Mercer, 2005a; Sweller et al., 2011). Nevertheless, it has been argued that “[i]nterpreting *via* video link has come to be seen as a more effective way of providing spoken language interpreting services than telephone interpreting” (Skinner et al., 2018, p. 13). In video-relayed DI, the image typically consists of the speaker, the podium, a panoramic view of the meeting room, or a partial view of the meeting room (Mouzourakis, 2003). However, in cloud-based meetings and conferences, only the speaker can be captured by the computer-embedded camera and/or the image on the screen with PowerPoint (PPT) slides. The image provided is determined by the angle of the feeding cameras, which might be microphone-activated or handled by multiple cameramen. In onsite SI, interpreters can make decisions about their own viewing behaviors to search for useful information to assist with the comprehension and production of language. Thus, the gaze of interpreters is a problem-driven, selective, and active process (Mouzourakis, 2006). In addition, research in language comprehension has shown that dividing attention between spatially and temporally segregated stimuli results in high cognitive load measured by proxies of gaze behavior and brain activity (Aryadoust et al., 2020b). This suggests that the separation of different sources of input in multimodal DI should be considered and investigated.

Materials and information specificity are pre-interpreting factors, which refer to the materials given to the interpreters before the conferences or meetings to facilitate their preparation (Kalina, 2005). Interpreters may receive PPT slides, speech drafts, or other supplementary materials before the conference (Díaz-Galaz 2011). Under such circumstances, these factors are expected to have a facilitating role in DI, thus probably reducing cognitive load.

Another task-related factor is turn duration in DI in simultaneous mode, which is suggested to be 20–30 min to avoid interpreter fatigue and possible decreases in interpreting quality (Kalina, 2002; Chmiel, 2008). However, there is no consensus over the turn duration in the general field of DI or distance consecutive interpreting. Despite the inadequacy of research on this factor, it can be surmised that physical and mental fatigue is likely to affect the quality of DI, and should therefore be taken into account in designing studies and in real-life DI.

In contrast with onsite interpreting, DI places the participants (i.e., interpreters, speakers and audience) in physically remote locations in computer-based collaborative environments, which may lead to less seamless participant interactivity. A growing body of evidence suggests that online environments can facilitate participant interactivity during interpreting (Arbaugh and Benbunan-Fich, 2007). In DI, especially in telephone-relayed interpreting, participant interactivity mainly includes turn-taking management, stopping the primary speakers(s) by using proper techniques to cut in, and seeking clarification (Wang, 2018). In simultaneous DI, where interpreters work with a partner or a team (in multilingual relay interpreting), physical separation from partners, clients, and technicians may affect performance in the distance mode. For instance, while onsite SI interpreters can maintain close contact with clients in a conference hall to acquire first-hand updates on conference procedures, DI interpreters cannot have physical proximity with the client (Davitti and Braun, 2020). Therefore, the remote mode of DI is likely to impede or minimize interactivity and limits the efficacy of communication (Licoppe and Veyrier, 2017; Powell et al., 2017). Mouzourakis (2003) believes that better technology equipment and more ergonomic arrangements of screens and monitors can mitigate this obstacle.

With regards to directionality, it has been recommended that SI interpreters should work from their L2 language (second language) to their L1 language to ensure quality (Seleskovitch and Lederer, 1989). However, scholars are still debating whether working in both directions is feasible, especially in the Chinese and Arabic languages based on empirical performance tests (i.e., Al-Salman and Al-Khanji, 2002; Chang and Schallert, 2007).

*Linguistic factors of the source input* entail the lexical, syntactic, semantic, textual, pragmatic, and sociolinguistic levels, and are common between onsite interpreting and DI (Hymes, 1972, 1974; Canale and Swain, 1980; Leung, 2005). At the lexical level, some types of vocabulary can result in a heavier cognitive load, specifically technical terminologies, numbers, and acronyms, since these words require domain-specific knowledge to process (Kalina, 2005; Gile, 2008). Semantic units, such as culturally loaded expressions or counterfactuals, might cause cognitive saturation in DSI (Setton, 2002; Vianna, 2005). Syntactic organization and complexity can also affect cognitive load in SI (Chmiel and Lijewska, 2019). For example, Seeber and Kerzel (2011) used eye-tracking to investigate the differences in the cognitive load of verb-initial (syntactically symmetrical) and verb-final (syntactically asymmetrical) structures during German-English SI, finding higher cognitive load with asymmetrical structures. In another study, Setton (1999, 2001, 2002) suggested that the pragmatic dimension, which is pertinent to the underlying message, can help build a mental model of SI for analyzing and demonstrating attitudes, intentions, and implications.

Sociolinguistic factors are related to language variety and register. For example, with English as a *lingua franca*—meaning that English is used as a common communicative means across different cultures—unique varieties of English have emerged in different parts of the world (e.g., Singlish, Indian English, etc.; Firth, 1996; Jenkins, 2000). Thus, an unfamiliar variety of linguistic codes might increase interpreters’ cognitive load. In fact, language policy research on how interpreters react to different varieties of language has gained much popularity among translation and interpreting researchers due to its strong influence in the 21st century (Zhu and Aryadoust, 2022).

*Paralinguistic characteristics of the source input* include delivery speed, accent, and body language of the speakers. Delivery speed is known to be a major factor affecting cognitive load in SI (Pio, 2003; Meuleman and Van Besien, 2009). Accent also contributes to cognitive load change in SI since it affects the comprehension of the input (Gile, 2009; Gluszek and Dovidio, 2010). Another factor is body language, which in DI is quite different from that in onsite SI where interpreters can see and thus utilize the body language and facial expression of speakers for useful information. However, due to the scale and arrangement of the interpreting booth, interpreters might not have a clear visual of the speakers. In DI, body language (if any, depending on the image, as discussed earlier) is delivered using digital technology. As a result, interpreters might have high-quality images of the speaker’s body language to use for paralinguistic help. However, at the time of writing this paper, whether DI digital images or onsite images influence interpreters’ cognitive load is unchartered waters requiring further study. Furthermore, the kind of digital images that may lower cognitive load, thus enhancing performance, is still open for discussion.

*Environment factors* include booth type, platform, physical parameters, transmission techniques, and contact with participants (Braun et al., 2018; AIIC, 2021). DSI interpreters might work in DSI hubs, home studios, or onsite booths that vary not only in their air quality, acoustics, ventilation, and temperature, but also differ in signal quality based on whether ethernet, WiFi, or mobile data is used for transmission (Moser-Mercer, 2003; Mouzourakis, 2006). For example, home-based studios may not have optimal acoustics since their location may be a home office or simply a desk in the corner of the interpreter’s house. Research shows that background noise affects interpreters’ stress levels, thus increasing cognitive load (Koskinen and Ruokonen, 2017). In a recent neuroimaging study, Lee et al. (2020) found that environmental and nature sounds evoke significantly different neurocognitive processes than long pieces of discourse. Similarly, it has been shown that noise could have adverse effects on language comprehension performance under experimental conditions (Fujita, 2021). This suggests that the concurrent presence of background noise and language input in DI can result in the additional activation of some brain regions and intensify the mental load due to interfering noise. In sum, physical environments can interact with task and interpreter characteristics, as predicted by instruction research on CLT (Choi et al., 2014).

Having described the task and environment variables in DI, we will now examine the operator’s (in this case, the DI interpreter) characteristics and possible moderating variables (Meshkati, 1988).

### Interpreter’s Factors in DI

In DI, interpreter factors include linguistic knowledge, topic knowledge, interpreting strategies, personal traits, technology awareness, and (meta-)cognitive processes. Skinner et al. (2018) states that “any modifications to interpreters’ working environments are likely to impact their performance and how they process information” (p. 19) since interpreting involves highly complex cognitive and metacognitive processes (Moser-Mercer, 2000; Gile, 2009). That is to say, the factors of the (meta-)cognitive processes are under the influence of all the other factors as indicated in Figure 2, which are explained in detail here.

The prerequisite for any DI interpreter to render professional services is *linguistic knowledge*, which is knowledge of the languages they work in (Kalina, 2000). The linguistic knowledge required in interpreting can be thought as “a domain of information in memory that is available for use” (Bachman and Palmer, 1996, p. 67). Linguistic knowledge is a component of the memory storage and consists of an array of competencies such as grammatical and discourse competence. It may be said that interpreters’ linguistic knowledge contributes to their capacity to perform more complex cognitive tasks (Choi et al., 2014). Then, if the tasks and other conditions remain the same, the interpreter with better linguistic knowledge can perform better since they can allocate their memory resources to other cognitive tasks involved in interpreting such as auditory and/or visual processing, long-term memory extraction or speech production monitoring.

There are four areas of linguistic knowledge in DI, similar to those described by Bachman and Palmer (1996): grammatical knowledge, textual knowledge, functional knowledge, and sociolinguistic knowledge. Grammatical knowledge concerns vocabulary, syntax, and phonology or graphology. Textual knowledge concerns cohesion, rhetorical use, and conversational organization. Functional knowledge involves the use of ideational functions (i.e., descriptions of happiness, explanations of sadness), manipulative functions (i.e., compliments, commands), heuristic functions (i.e., teaching, problem-solving), and imaginative functions (i.e., jokes, novels). Sociolinguistic knowledge is knowledge of dialects, registers, idiomatic expressions, culture-loaded references, and figures of speech. Numerous studies have shown that linguistic knowledge is directly proportional to the cognitive load, thus affecting their performance (Gile, 1995; Angelelli and Degueldre, 2002).

*Topic knowledge* of cultures of various countries or regions is another key factor in DI. This includes knowledge of the administrative structures of both sides of participants, political, economic, social, and ethnic characteristics of the participants’ areas of origin, and even literature and the arts (Kalina, 2000).

Topic knowledge has always been recognized as a powerful predictor of comprehension in both expert and novice groups since it interacts with text structure and verbal ability at the micro level while engaging in the metacognitive strategy use at the macro level (McNamara et al., 2007; Díaz-Galaz et al., 2015). That is, interpreters use the topic knowledge to integrate the information presented in the source speech in a continuous way to construct a mental representation, which they subsequently reformulate and articulate in the target language.

In recent years, *personal traits*, also termed personal psycho-affective factors, such as motivation, anxiety, and stress resistance, have also been recognized as important components of an interpreter’s aptitude (Bontempo and Napier, 2011; Rosiers et al., 2011; Timarová and Salaets, 2011). For example, Korpal (2016) measured heart rate and blood pressure to determine whether the speed of a speaker’s delivery influenced the interpreter’s stress level. Heart rate, but not systolic or diastolic blood pressure indices, was significantly associated with speech rate, supporting the assumption that a faster speech rate can make interpreters experience higher levels of stress. In DI, several studies have examined stress and burnout, finding that the DI interpreters experience high levels of stress and burnout (Roziner and Shlesinger, 2010; Bower, 2015). A study by Seeber et al. (2019) suggested that providing appropriate visual input is important to alleviate the alienation of DI interpreters and a study by Ko (2006) concluded that longitudinal empirical studies are an essential methodology in DI research. The research methods used to investigate personal traits are becoming increasingly diverse, ranging from qualitative surveys to objective psycho-physical instruments (e.g., eye-tracking).

*Interpreting strategies* are derived from the understanding of the underlying processes of interpreting, and can help interpreters to apply the optimal interpreting solutions across communicative settings (Riccardi, 2005). These strategies refer to the skills needed to comprehend and produce language, which might be included among or in addition to the strategies used in monolingual language processing. Notably, interpreters (i) are not expected to alter or filter out information, and (ii) may not have sufficient domain knowledge (Riccardi, 1998, 2005; Kalina, 2000; Shlesinger, 2000; Chang and Schallert, 2007).

Given that DI is a technology-supported language mediation method, *technology awareness* is an important interpreter factor that affects cognitive load. Compared to translation, where computer-assisted services have become standard, interpreting has experienced only a modest impact due to advances in information and communication technology (Fantinuoli, 2018). This is because voice recognition technologies cannot fully recognize natural spoken language and its hesitations, repairs, hedges, and unfinished sequences (Desmet et al., 2018). Nevertheless, technology can be used to support DI in multiple ways.

Technology awareness in DI comprises two dimensions: (i) being mindful that technology is an inevitable part of the interpreting industry and being ready to accept it; and (ii) being able to recognize and utilize technology to perform DI (Parsons et al., 2014). For example, it is well known that numbers are difficult to interpret and consume tremendous cognitive resources. To support the translation of numbers, Desmet et al. (2018) used booth technology to automatically recognize numbers in source speech and present them on a screen, which significantly enhanced translation accuracy. Similarly, Prandi (2018) explored the use of computer-assisted interpreting tools to manage terminology, aiming to reduce local cognitive load during terminology search when delivering the target text. With new information and tools emerging due to technological advances, DI interpreters need to be fully prepared to reap the advantages and opportunities of technology while minimizing the risks and consequences that arise from their use.

Mellinger and Hanson (2018) pointed out that the intersection between technology and interpreting remains “under-theorized,” particularly regarding the adaptation of technology in accomplishing the interpreting task. They conducted a survey-based investigation to examine the self-perception of the interpreters’ role in technology use and adoption. They found that community interpreters in medical and court settings where DI first appeared and achieved acceptance are more likely to adopt new technologies than their counterparts in conference settings. Few empirical studies have hitherto examined interpreters’ technology awareness, thus leaving a big gap in the understanding of the use of technology in DI.

*Cognitive process* is the ability to build up a mental representation of the verbal message through comprehension, parsing the information, and integrating it with one’s own pre-existing knowledge (Setton, 1999). *Metacognitive processes*, on the other hand, refer to strategies for efficient management of processing resources and consist of planning, monitoring, and evaluating (Flavell, 1976). Bravo (2019) reviewed metacognition research in interpreting and concluded that “[m]onolingual communication requires a lesser degree of metacognitive awareness than interpreter-mediated communicative events do” (p. 148). This is due to the fact that the nature of the interpreting task, which demands the ability to quickly shift attention across many cognitive activities, needs a meta-level skill to perform quality control. Through applying proper cognitive processes and metacognitive strategies, interpreters interact with the task and environment factors, a process that influences interpreters’ performance.

So far, we have looked into the factors which induce change in cognitive load during the interpreting process and categorized them as causal factors consisting of task and environment factors and interpreter factors. These factors act as the proxies representing dimensions of cognitive load. The methods for measuring these factors to assess the cognitive load that DI interpreters experience are discussed in the following section.

## Measurement Methods

Cognitive load in DI is conceptualized as a multidimensional construct representing the load that performing a particular interpreting task imposes on the distance interpreter’s cognitive system (Paas and Van Merriënboer, 1994; Seeber, 2013). To measure this construct, “the most appropriate measurement techniques” should be used (Meshkati, 1988, p. 306). Scholars have attempted to model and measure cognitive load with various methods (DeLeeuw and Mayer, 2008; Wang et al., 2020; Ayres et al., 2021; Krell et al., 2022; Ouwehand et al., 2022). In this study, we adopt a comprehensive and fine-grained categorization of Paas et al. (2003) and Schultheis and Jameson (2004) who proposed four discrete methods for measuring cognitive load: subjective methods, analytical methods, performance methods, and psycho-physiological methods. This section provides a review of the pros and cons of each of these measurement methods and how they can be used to investigate specific DI factors. Some of the pioneering studies utilizing these measurement methods in DI are presented in Table 2. These measurement methods, despite having been used and verified in interpreting studies for quite some time, have not been widely used in DI research. Therefore, the following discussion of these methods will largely rely on their previous application in interpreting studies.

**Table 2:** Measurement methods of cognitive load in DI.

Measurement methods	Pioneering studies in DI	The specific factors under investigation
Subjective methods	Mouzourakis (2006); Bower (2015)	Stress and burnout; Sound and image transmission in the environment
Analytical methods	Braun (2007)	Cognitive process of the interpreter
Performance methods	Jiménez-Ivars (2021); Braun (2013); Gany et al. (2007)	Psycho-affective factors of the interpreters; Distance working mode; Distance working mode
Psycho-physiological methods	Kuang and Zheng (2022); Roziner and Shlesinger (2010)	Source speech difficulty and interpreters’ experience; Mode of presentation of the DI task

### Subjective Methods

Subjective methods of measuring cognitive load in DI—such as Likert scales and verbal elicitations and reports—require participants to directly estimate or compare the cognitive load they experienced during a specific task at a given moment (Reid and Nygren, 1988). Subjective methods of cognitive load are based on the assumption that participants are able to recall their cognitive processes and report the amount of mental effort required, which is a limitation that researchers should be aware of (Ericsson and Simon, 1998). Subjective measures enjoyed popularity in early research because they are easy to use, non-intrusive, low-cost, and can discriminate between different load conditions (Luximon and Goonetilleke, 2001).

In interpreting studies, subjective methods can provide data on: (i) how interpreters allocate attention; (ii) problem-solving strategies used by interpreters; (iii) the effect of interpreting expertise; and (iv) general assessment of cognitive activities in interpreting (i.e., comprehension, translation, and production; Ivanova, 2000). In early studies of DI, subjective methods were used to investigate how transmission techniques are used by interpreters (Böcker and Anderson, 1993; Mouzourakis, 1996, 2006; Jones et al., 2003). Among them, Mouzourakis (2006) reviewed the large-scale DI experiments that were conducted at the United Nations and the European Union institutions in which the subjective data collected by questionnaire were used to indicate how the technical setup for sound and image transmission would impact interpreters’ perceptions of DI. Later, this method was also adopted in DI studies to evaluate interpreters’ stress levels (Ko, 2006; Bower, 2015; Costa et al., 2020), the effect of interpreters’ visibility on participants (Licoppe and Veyrier, 2017), turn-taking (Davitti and Braun, 2020; Havelka, 2020), and the effect of different presentation modalities (Braun, 2007).

However, Lamberger-Felber (2001) performed a comparative study of objective text presentation parameters and interpreters’ subjective evaluations of texts, finding differences for almost all parameters investigated. This caution is important because subjective judgments usually serve as the method (or part of the method) used to assess the difficulty of instruments (e.g., Su and Li, 2019; Weng et al., 2022) together with more ‘objective’ measures (e.g., using textual analysis to measure the difficulty level of the source text). Seeber (2013) acknowledged that subjective methods might not be able to “reliably assess cognitive load” (p. 8) in SI.

Chandler (2018) cautioned that subjective methods are limited due to their dependence on participants’ self-appraisal and personal judgment as well as being problematic with young children. For example, Low and Aryadoust (2021) found that listeners’ self-reports of strategies had a significantly lower predictive power in accounting for oral comprehension performance compared with gaze behavior measures collected through an eye tracker. In the general field of cognitive load measurements, an instrument is usually developed and validated in one study, and further validated in other studies under different conditions. A typical example is that the cognitive load scale (CLS) developed by Leppink et al. (2013) was widely used as a subjective measurement tool of three types of cognitive load at large, and was further validated and expanded by Andersen and Makransky (2021) to measure the cognitive load for physical and online lectures. In DI, the lack of internal reliability of the instruments is a noteworthy concern, since the instruments are mostly formulated by the investigators to answer their own specific questions, and therefore require further validation. Recognizing these limitations, Ayres et al. (2021) proposed that the combination of subjective and physiological measures is most effective in investigating change in cognitive load. Therefore, we recommend that the results of the studies that use subjective methods for data collection be cross-validated with objective techniques such as eye-tracking and neuroimaging (see Aryadoust et al., 2020b, for an example of measuring cognitive load using eye-tracking and brain imaging in comprehension tasks). In addition, appropriate reliability checks should be applied to ensure the precision and replicability of subjective methods of measuring change in cognitive load in DI (Moser-Mercer, 2000; Riccardi, 2005; Fantinuoli, 2018).

### Performance Methods

Performance methods of measuring cognitive load have long been used in interpreting studies to measure speed and accuracy. For example, calculation of the ear-to-voice span (EVS) by Oléron and Nanpon (1965) probed cognitive processing of interpreters’ performance *via* quantitative measures. Later, Barik (1973) compared the performance of professionals, interpreting students, and bilinguals without any interpreting experience by counting the errors, omissions, and additions in their output. This tradition of comparing the performance of participants with different levels of expertise has carried on until the present day in investigating cognitive behavior. Given that participants’ performances may have been evaluated by human raters with different leniency and severity, modern psychometric methods like the many-facet Rasch measurement (MFRM) have been used to validate rating scales and identify the degree of severity/leniency of raters (Han, 2015, unpublished Doctoral dissertation)^2^.

Performance methods are also used to compare the different tasks concerning language comprehension and production. For example, Christoffels and De Groot (2004) designed an experiment comparing SI, paraphrasing, and shadow sentences (repeating), with the latter two considered to be delayed conditions. The authors assumed that participants would perform better in the delayed conditions than in SI, since simultaneous comprehension and production are a major cause of difficulty in SI. The results showed that the poorest performance was for SI, followed by paraphrasing and then repeating, which indicated the increased cognitive load of SI compared to the other two tasks.

In DI, performance methods have been widely used to compare the quality of onsite interpreting along with interpreting in different distance modes (Oviatt and Cohen, 1992; Wadensjö, 1999; Moser-Mercer, 2005a,b; Gany et al., 2007; Locatis et al., 2010; Braun, 2013, 2017; Jiménez-Ivars, 2021). For example, Gany et al. (2007) investigated how various interpreting methods affect medical interpreters’ speed and errors through comparing their DI performance with onsite interpreting, finding that the DI mode resulted in fewer errors and was faster. Combined with interpreters’ subjective perceptions of their performances, objective performance measures have helped to increase the acceptance of DI. However, previous studies are not directly comparable with each other since they were set up under different circumstances and interpreting modes, making the further evaluation of DI quality necessary (Braun, 2020).

For measuring cognitive load in DI, it is suggested that performance measures be used along with other methods to investigate factors like interpreters’ overall performance, interpreters’ linguistic knowledge, and topic knowledge (Mazza, 2001; Gile, 2008; Timarová and Salaets, 2011).

### Analytical Methods

Analytical methods use expert opinions and mathematical models to estimate cognitive load in DI and interpreting at large (Paas et al., 2003). Psychologists proposed an analytical and empirical framework to accommodate the measurement of cognitive load (e.g., Linton et al., 1989; Xie and Salvendy, 2000). Following their lead, early interpreting scholars proposed several cognitive models to explain the cognitive processes involved in interpreting (Gerver, 1975, 1976; Moser, 1978; Gile, 1995, 2009; Mizuno, 2005; Seeber, 2011; Ketola, 2015).

Gerver’s (1975, 1976) model focused on the short-term storage of the source text, which stays in a hypothetical input buffer in the mind from which the source text is sent out for further processing. The processing is performed in cooperation with long-term memory, which activates pertinent linguistic units for externalization *via* an output buffer. It is quite a modern idea that Gerver (1976) described information from several sources being processed in parallel. However, the separate input and output buffers still lack any theoretical or empirical support (Timarová, 2008).

Moser (1978) proposed another cognitive model of SI that assigns a crucial role to WM, which she instead called generated abstract memory (GAM). In this model, WM is conceptualized as a structural and functional unit that stores processed chunks (the STM storage function), performs comprehension tasks in cooperation with long-term memory (the executive functions), and plays a role in production as well (see Moser, 1978, p. 355 for details). According to Moser-Mercer (1997), the model also contains a prediction step for incoming content which, she argues, plays a crucial role in interpreting.

An alternative cognitive model of SI was proposed by Darò and Fabbro (1994). This model is based on the models of Baddeley and Hitch (1974) and Baddeley (1992) but only adopts the verbal slave system and central executive elements. Darò and Fabbro (1994) explicitly separate the cognitive process into two general processes: WM and long-term memory processes. Interestingly, in this model, translation in two directions is performed by two separate mental modules, which are the basis for investigation of directionality effects in future studies (See Darò and Fabbro, 1994, p. 381, for model details).

Gile’s (1995, 2009) effort model and Seeber’s (2011) cognitive load model are two milestone analytical models in measuring cognitive load. These models were extensively used as a means of understanding cognitive load in SI and are discussed in terms of the measurement of cognitive load in the current study. Both models provide useful insights for capturing the complex multi-tasking process in SI. However, Seeber (2013) acknowledges that both models are unable to account for individual differences, which is a major constraint for measuring cognitive load in interpreting. For example, due to individual differences in EVS in SI, it is difficult to establish a cause-and-effect relationship between cognitive load, performance quality, and performance speed (Seeber, 2013).

Braun and her colleagues attempted to establish several analytical frameworks to assess DI interpreters’ cognitive load generated across different settings (Braun, 2007, 2017; Braun and Taylor, 2012). For example, Braun (2007) used a process-oriented model of communication in which linguistic and cognitive processes contribute to discourse comprehension and production. Braun managed to investigate the adaptation process of the DI interpreters. Using a microanalytical framework, Davitti and Braun (2020) drew on extracts from a corpus of DI encounters to identify the coping strategies in online collaborative contexts, which include managing turn-taking, spatial organization, and the opening and closing of a DI encounter. For example, by analyzing the spatial organization behavior of interpreters, they found that explicit instructions from DI interpreters can create more mutual visibility and awareness to ease their mental load and support their performance. Overall, the study provides substantial implications for interpreter education.

Analytical methods in interpreting can be mainly used to evaluate input dimensions of cognitive load, meaning that researchers can apply judgment and/or mathematical methods to measure the cognitive load induced by the task and environment factors (Chen, 2017; Ehrensberger-Dow et al., 2020). Although a fine-grained analytical model of the overall cognitive load in DI is still to be developed, we believe the current study would be a step toward specifying the key causal factors of cognitive load in DI and their measurement methods.

### Psycho-Physiological Measures

Based on the assumption that psycho-physiological variables covary with cognitive load, psycho-physiological measures have been used in the investigation of cognitive load, such as changes in heart, brain, skin, or eye responses (Paas et al., 2003; Schultheis and Jameson, 2004). Psycho-physiological measures can provide direct and continuous data in DI, allowing online measurement with a high sampling rate and sensitivity without interference from additional task(s). These measures are particularly useful for probing the “black box” of interpreters, that is, their cognitive process (Seeber, 2013). Brain imagining, stress hormones, eye-tracking, and galvanic skin response (GSR), which are widely used methods in measuring cognitive load, are discussed here.

#### Brain Imaging

Brain imaging can provide a “window” to examine cognitive load in interpreting. Kurz (1994, 1995) used electroencephalography (EEG) to investigate the effect of directionality during shadowing and SI tasks. Price et al. (1999) and Rinne et al. (2000) both used positron emission tomography (PET) to examine brain activation during interpreting, finding pronounced bilateral activation of the cerebellum and temporal and frontal regions during the assigned tasks. Tymoczko (2012) pointed out that using technology and neuroscience in interpreting research is “one of the most important known unknowns of the discipline” (p. 98). Recent research (e.g., Elmer et al., 2014; Becker et al., 2016; Hervais-Adelman et al., 2018; Van de Putte et al., 2018; Zheng et al., 2020) has continued to use the functional magnetic resonance imaging (fMRI) method of brain imaging to better understand interpreting (See a comprehensive review of brain imaging in interpreting studies in Muñoz et al., 2018). Of course, a caveat concerning the use of fMRI is that it reduces the ecological validity of experiments. Recent neuroimaging techniques such as functional near-infrared spectroscopy (fNIRS), which allow for more mobility and maintain ecological validity, are recommended as alternative measurement methods (Aryadoust et al., 2020a).

#### The Stress Hormone Cortisol

The level of the stress hormone cortisol is a psycho-physiological measure that has been used in DI research. Roziner and Shlesinger (2010) sampled the interpreters’ saliva four times a day for the cortisol levels, and compared these indices between the onsite and distance modes, finding that the mean cortisol levels of interpreters in distance modes were slightly higher than that in onsite modes in working hours. For future studies, different indicators of stress level (e.g., blood pressure, heart rate, GSR) can be measured together for a better understanding of the phenomenon.

#### Eye-Tracking

Among psycho-physiological techniques, eye-tracking has long attracted the interest of interpreting researchers given that our eye-movements, or gaze behavior, can reflect the continuous processes in our mind (Hyönä et al., 1995; Dragsted and Hansen, 2009; Chen, 2020; Tiselius and Sneed, 2020; Amos et al., 2022; Kuang and Zheng, 2022). Specifically, eye-tracking is noninvasive and has proved to be useful for describing “how the interpreter utilizes his or her processing resources, and what factors affect the real-time performance” (Hyönä et al., 1995, p. 8). In recent years, eye-tracking has been applied in various studies to investigate how cognitive load in the interpreting process is affected by factors including syntactic characteristics of source speech (Seeber et al., 2020), delivery rate (Korpal and Stachowiak-Szymczak, 2020), interpreting strategies (Vranjes et al., 2018a,b), and cognitive effort (Su and Li, 2019). A detailed review of the application of eye-tracking in investigating cognitive load in interpreting research is reported by Zhu and Aryadoust (under review).

#### Galvanic Skin Response

In recent years, GSR, a marker of emotional arousal, has also attracted attention in interpreting studies (Korpal and Jasielska, 2019). In research using GSR, it is assumed that physiological arousal activates the sympathetic nervous system (SNS), resulting in more pronounced skin conductance.

Even though they require a complex and refined experimental design, psycho-physiological measures appear to be a promising set of methods for measuring real-time or delayed cognitive load during the interpreting process (Seeber and Kerzel, 2011). The task and environment factors discussed earlier induce a certain level of cognitive load, thus leading to physiological changes in interpreters. As a result, interpreters are ideal subjects for physiological measures. Given their promising applications in DI, the question is not whether psycho-physiological measures should be used, but how to control the variables to investigate what is really being estimated (the construct itself; Seeber, 2013).

In summary, the four aforementioned methods of measuring cognitive load in DI each have their own unique strengths and weaknesses. Given that the empirical research on DI started two decades ago, these measurement methods have not been widely applied in DI research. The studies we surveyed above provide examples for future researchers to design their studies. Furthermore, the use of these methods in the broader interpreting field can provide a link between previous interpreting studies and future DI studies. These studies can be replicated in the field of DI to examine how technological challenges and remoteness may alter cognitive complexity and difficulty in interpreting (Braun, 2007), thus providing research directions for interpreting practitioners and trainers. Researchers should consider their research aims and variables under investigation to determine the most appropriate measure types for their study.

## Toward a Cohesive Representation of Cognitive Load in DI

This paper has discussed cognitive load theory and the general cognitive models in interpreting. It has also presented a discussion of the causal factors inducing change in cognitive load in DI and their measurement methods. These discussions were intended to present a scientific representation to bridge the abstract concept of cognitive load and the world experienced in DI practice and research. As a first attempt to integrate representations of cognitive load and measurement methods in DI, this current discussion offers several important implications related to DI.

First, this synthesis review provides a multicomponential representation of cognitive load in an endeavor to concretize this abstract concept (Huddle et al., 2000). As previously discussed, the factors that change cognitive load play the role of surrogates for it, but they are scattered across previous studies on cognitive load in interpreting. This paper sought to synthesize them into two general categories (i.e., task and environment factors, and DI interpreter factors) and then unpack the categories to make them more accessible to interested researchers. Informed by CLT, the identified causal factors constitute our endeavor to assist the stakeholders (e.g., conference organizers and training program managers) in controlling for extraneous factors and directing mental resources of interpreters to intrinsic and germane sources of cognitive load. The study also provides a framework for future experimental designs to control confounding variables and optimize research design by identifying influential variables in DI and their relationship. For example, in terms of experimentation for identifying causal relationships between DI interpreters’ performance and mode of presentation (e.g., video-relayed or telephone interpreting), researchers should control for confounding variables like source speech complexity and participants’ proficiency level to be able to establish causality. Our discussion of the causal factors could be used as a checklist for the experiment designers in DI or even interpreting studies in general.

Second, we identified the factors that can affect cognitive load in DI and reviewed the relevant literature in interpreting studies in general and DI in particular, which makes the current study distinct from past discussions of interpreting which tend to be broad and general. Accordingly, the present synthesis may also serve as the basis for future replication studies in DI. This is in line with Rojo López and Martín (2022) who suggested that replication studies are required to foster the standardization of general research methodology in studying the cognition behavior of interpreters. Since technology has played an increasingly important role in the interpreting service industry, the factors specified for DI in this study would provide important implications for research and practice. For example, the identification of technology awareness factors would help curriculum designers and trainers in translation and interpreting programs to embed these factors into course contents (Ehrensberger-Dow et al., 2020).

Third, the framework presented in this paper serves not only as a descriptor of factors affecting cognitive load, but also as a predictive tool in which the pros and cons of the measurement methods were analyzed and presented (Chittleborough and Treagust, 2009). Thus, it can help researchers actively make plausible predictions and informed decisions in study design, e.g., by choosing appropriate measurement methods to conduct investigations and controlling for possible extraneous variables. As earlier noted, subjective measurement methods such as interviews or surveys can help provide *post hoc* evaluations of cognitive load in DI, while performance measures provide indications of interpreting proficiency based on objective and/or subjective ratings. Analytical measures, on the other hand, provide an estimate of cognitive load based on subjective and analytical data, thus relying on the prior knowledge of the investigators (Seeber, 2013). Finally, psycho-physiological measures register detailed real-time patterns of cognitive activity, and require a well-controlled experimental design which is not confounded by construct-irrelevant causal variables.

We note that the current discussion is based mainly on researchers’ understanding of cognitive load in DI and interpreting studies in general, which makes it an expressed model. An expressed model needs to be tested by empirical studies and agreed upon by society, to become a consensus model (Gilbert, 2004). Therefore, we call for future empirical studies to validate this representation with recommended methods and expand the scope of these methods to examine the factors contributing to cognitive load in DI.

## Author Contributions

XZ and VA contributed equally to the conceptualization, writing, and revisions of the article and approved the submitted version.

## Funding

This research was supported by a research fund from Sichuan International Studies University (SISU), China (Grant No: SISUWYJY201919).

## Conflict of Interest

The authors declare that the research was conducted in the absence of any commercial or financial relationships that could be construed as a potential conflict of interest.

## Publisher’s Note

All claims expressed in this article are solely those of the authors and do not necessarily represent those of their affiliated organizations, or those of the publisher, the editors and the reviewers. Any product that may be evaluated in this article, or claim that may be made by its manufacturer, is not guaranteed or endorsed by the publisher.

## Footnotes

^1^Meshkati, N. (1983). *A conceptual model for the assessment of mental workload based upon individual decision styles.* [Unpublished Doctoral dissertation]. University of Southern California.

^2^Han, C. (2015). *Building the validity foundation for interpreter certification performance testing.* [Unpublished Doctoral dissertation]. Macquarie University.

## References

[ref2] AIIC (2020a). Covid-19 Distance nterpreting Recommendations for Institutions and DI Hubs. Available at: https://aiic.org/document/4839/AIIC%20Recommendations%20for%20Institutions_27.03.2020.pdf (Accessed May 30, 2021).

[ref1] AIIC (2020b). AIIC Guidelines for Distance Interpreting (Version 1.0). Available at: https://aiic.org/document/4418/AIIC%20Guidelines%20for%20Distance%20Interpreting%20Version%201.0 (Accessed May 30, 2021).

[ref3] AIIC (2021). AIIC and distance interpreting. Available at: https://aiic.org/site/world/about/profession/distanceinterpreting (Accessed May 30, 2021).

[ref4] AIIC (2022). AIIC position on distance interpreting. Available at: https://aiic.org/document/1031/AIIC-Position-on-Distance-Interpreting-05032018.pdf (Accessed February 15, 2022).

[ref501] Ait Ammour H. (2021). Onsite interpreting versus remote interpreting in the COVID 19 world. Rev. Appl. Linguis. 5, 339–344. PMID: 4768192

[ref5] Al-Salman S. Al-Khanji R. I. (2002). The native language factor in simultaneous interpretation in an Arabic/English context. Meta 47, 607–626. doi: 10.7202/008040ar

[ref6] Amos R. M. Seeber K. G. Pickering M. J. (2022). Prediction during simultaneous interpreting: evidence from the visual-world paradigm. Cognition 220:104987. doi: 10.1016/j.cognition.2021.104987, PMID: 34922159

[ref7] Andersen M. S. Makransky G. (2021). The validation and further development of the multidimensional cognitive load scale for physical and online lectures (MCLS-POL). Front. Psychol. 12:642084. doi: 10.3389/fpsyg.2021.642084, PMID: 33815228 PMC8014070

[ref8] Angelelli C. Degueldre C. (2002). “Bridging the gap between language for general purposes and language for work: An intensive superior level language/skill course for teachers, translators, and interpreters,” in Developing Professional-Level Language Proficiency. eds. Leaver B. L. Shekhtman B. (Cambridge: Cambridge University Press), 91–110.

[ref700] Arbaugh J. B. Benbunan-Fich R. (2007). The importance of participant interaction in online environments. Decision Support Syst. 43, 853–865. doi: 10.1016/j.dss.2006.12.013, PMID: 33815228

[ref9] Aryadoust V. Foo S. Ng L. Y. (2020a). What can gaze behaviors, neuroimaging data, and test scores tell us about test method effects and cognitive load in listening assessments? Lang. Test. 39, 56–89. doi: 10.1177/02655322211026876

[ref10] Aryadoust V. Ng L. Y. Foo S. Esposito G. (2020b). A neurocognitive investigation of test methods and gender effects in listening assessment. Comput. Assist. Lang. Learn. 35, 743–763. doi: 10.1080/09588221.2020.1744667

[ref11] Ayres P. Lee J. Y. Paas F. van Merriënboer J. J. (2021). The validity of physiological measures to identify differences in intrinsic cognitive load. Front. Psychol. 12:702538. doi: 10.3389/fpsyg.2021.702538, PMID: 34566780 PMC8461231

[ref12] Azarmina P. Wallace P. (2005). Remote interpretation in medical encounters: a systematic review. J. Telemed. Telecare 11, 140–145. doi: 10.1258/1357633053688679, PMID: 15901441

[ref13] Bachman L. F. Palmer A. S. (1996). Language Testing in Practice. Oxford: Oxford University Press.

[ref001] Baddeley A. D. (1986). Working memory. Oxford: Oxford University Press.

[ref14] Baddeley A. D. (1992). Working memory. Science 255, 556–559. doi: 10.1126/science.1736359 1736359

[ref16] Baddeley A. D. (2000). The episodic buffer: a new component of working memory? Trends Cogn. Sci. 4, 417–423. doi: 10.1016/S1364-6613(00)01538-2, PMID: 11058819

[ref002] Baddeley A. D. (2012). Working memory: theories, models, and controversies. Annu. Rev. Psychol. 63, 1–29. doi: 10.1146/annurev-psych-120710-100422 21961947

[ref17] Baddeley A. D. Hitch G. (1974). “Working memory,” in The Psychology of Learning and Motivation. ed. Bower G. A. (New York, NY: Academic Press), 47–89.

[ref18] Bae M. Jeong C. J. (2021). The role of working memory capacity in interpreting performance: an exploratory study with student interpreters. Transl. Cognit. Behav. 4, 26–46. doi: 10.1075/tcb.00050.bae

[ref20] Barik H. C. (1973). Simultaneous interpretation: temporal and quantitative data. Lang. Speech 16, 237–270. doi: 10.1177/002383097301600307, PMID: 4768192

[ref21] Becker M. Schubert T. Strobach T. Gallinat J. Kühn S. (2016). Simultaneous interpreters vs. professional multilingual controls: group differences in cognitive control as well as brain structure and function. NeuroImage 134, 250–260. doi: 10.1016/j.neuroimage.2016.03.079, PMID: 27085505

[ref23] Böcker M. Anderson D. (1993). “Remote conference interpreting using ISDN videotelephone: a requirements analysis and feasibility study,” in Proceedings of the Human Factors and Ergonomics Society Annual Meeting, Vol. 37 (Thousand Oaks, CA: SAGE Publications), 235–239.

[ref24] Bontempo K. Napier J. (2011). Evaluating emotional stability as a predictor of interpreter competence and aptitude for interpreting. Interpreting 13, 85–105. doi: 10.1075/intp.13.1.06bon

[ref25] Bower K. (2015). Stress and burnout in video relay service (VRS) interpreting. J. Interpretation 24:2

[ref26] Braun S. (2007). Interpreting in small-group bilingual videoconferences: challenges and adaptation processes. Interpreting 9, 21–46. doi: 10.1075/intp.9.1.03bra

[ref27] Braun S. (2013). Keep your distance? Remote interpreting in legal proceedings: a critical assessment of a growing practice1. Interpreting 15, 200–228. doi: 10.1075/intp.15.2.03bra

[ref28] Braun S. (2015). “Remote interpreting,” in Routledge Handbook of Interpreting. eds. Mikkelson H. Jourdenais R. (London: Routledge), 352–367.

[ref29] Braun S. (2017). What a micro-analytical investigation of additions and expansions in remote interpreting can tell us about interpreters’ participation in a shared virtual space. J. Pragmat. 107, 165–177. doi: 10.1016/j.pragma.2016.09.011

[ref30] Braun S. (2020). “Technology, interpreting,” in Routledge Encyclopedia of Translation Studies. eds. Baker M. Saldanha G. (London: Routledge), 569–574.

[ref31] Braun S. Davitti E. Dicerto S. (2018). “Video-mediated interpreting in legal settings: assessing the implementation,” in Here or There: Research on Interpreting via video link. eds. Napier J. Skinner R. Braun S. (Washington, DC: Gallaudet University Press), 144–179.

[ref32] Braun S. Taylor J. (2012). “Video-mediated interpreting: an overview of current practice and research,” in Videoconference and Remote Interpreting in Criminal Proceedings. eds. Braun S. Taylor J. (Antwerp: Intersentia), 33–68.

[ref0012] Bravo E. A. F. (2019). Metacognitive self-perception in interpreting. Translation, Cognition & Behavior 2, 147–164. doi: 10.1075/tcb.00025.fer

[ref34] Byrne A. (2013). Mental workload as a key factor in clinical decision making. Adv. Health Sci. Educ. 18, 537–545. doi: 10.1007/s10459-012-9360-5, PMID: 22411354

[ref35] Campbell S. Hale S. (2003). “Translation and interpreting assessment in the context of educational measurement,” in Translation Today: Trends and Perspectives. eds. Anderman G. M. Rogers M. (Clevedon: Multilingual Matters), 205–224.

[ref36] Cañada M. D. Arumí M. (2012). Self-regulating activity: use of metacognitive guides in the interpreting classroom. Educ. Res. Eval. 18, 245–264. doi: 10.1080/13803611.2012.661934

[ref37] Canale M. Swain M. (1980). Theoretical bases of communicative approaches to second language teaching and testing. Appl. Linguis. 1, 1–47. doi: 10.1093/applin/1.1.1

[ref38] Chandler P. (2018). “Forward,” in Cognitive load Measurement and Application: A Theoretical Framework for Meaningful Research and Practice. ed. Zheng R. Z. (London: Routledge), viii–x.

[ref39] Chang C. Schallert D. L. (2007). The impact of directionality on Chinese/English simultaneous interpreting. Interpreting 9, 137–176. doi: 10.1075/intp.9.2.02cha

[ref40] Chen S. (2017). The construct of cognitive load in interpreting and its measurement. Perspectives 25, 640–657. doi: 10.1080/0907676X.2016.1278026

[ref003] Chen S. (2018). A pen-eye-voice approach towards the process of note-taking and consecutive interpreting: An experimental design. Int. J. Comp. Lit. Translat. Stud. 6, 1–6. doi: 10.7575/aiac.ijclts.v.6n.2p.1

[ref41] Chen S. (2020). The impact of directionality on the process and product in consecutive interpreting between Chinese and English: evidence from pen recording and eye tracking. J. Specialised Transl. 34, 100–117.

[ref42] Chiang Y. N. (2009). Foreign language anxiety in Taiwanese student interpreters. Meta 54, 605–621. doi: 10.7202/038318ar

[ref43] Chittleborough G. D. Treagust D. F. (2009). Why models are advantageous to learning science. Educación química 20, 12–17. doi: 10.1016/S0187-893X(18)30003-X

[ref44] Chmiel A. (2008). Boothmates forever?—On teamwork in a simultaneous interpreting booth. Across Lang. Cult. 9, 261–276. doi: 10.1556/Acr.9.2008.2.6

[ref45] Chmiel A. (2018). In search of the working memory advantage in conference interpreting–training, experience and task effects. Int. J. Biling. 22, 371–384. doi: 10.1177/1367006916681082

[ref46] Chmiel A. Janikowski P. Lijewska A. (2020). Multimodal processing in simultaneous interpreting with text: interpreters focus more on the visual than the auditory modality. Targets 32, 37–58. doi: 10.1075/target.18157.chm

[ref47] Chmiel A. Lijewska A. (2019). Syntactic processing in sight translation by professional and trainee interpreters: professionals are more time-efficient while trainees view the source text less. Targets 31, 378–397. doi: 10.1075/target.18091.chm

[ref48] Choi H. H. Van Merriënboer J. J. Paas F. (2014). Effects of the physical environment on cognitive load and learning: towards a new model of cognitive load. Educ. Psychol. Rev. 26, 225–244. doi: 10.1007/s10648-014-9262-6

[ref49] Christoffels I. K. De Groot A. M. (2004). Components of simultaneous interpreting: comparing interpreting with shadowing and paraphrasing. Bilingualism 7, 227–240. doi: 10.1017/S1366728904001609

[ref50] Christoffels I. De Groot A. (2009). Simultaneous Interpreting. Handbook of Bilingualism: Psycholinguistic Approaches (pp. 454–479). Oxford: Oxford University Press.

[ref51] Cierniak G. Scheiter K. Gerjets P. (2009). Explaining the split-attention effect: is the reduction of extraneous cognitive load accompanied by an increase in germane cognitive load? Comput. Hum. Behav. 25, 315–324. doi: 10.1016/j.chb.2008.12.020

[ref52] Collard C. Defrancq B. (2019). Predictors of ear-voice span, a corpus-based study with special reference to sex. Perspectives 27, 431–454. doi: 10.1080/0907676X.2018.1553199

[ref53] Costa B. Gutiérrez R. L. Rausch T. (2020). Self-care as an ethical responsibility: A pilot study on support provision for interpreters in human crises. Transl. Interpreting Stud. 15, 36–56. doi: 10.1075/tis.20004.cos

[ref54] Darò V. Fabbro F. (1994). Verbal memory during simultaneous interpretation: effects of phonological interference. Appl. Linguis. 15, 365–381. doi: 10.1093/applin/15.4.365

[ref55] Davitti E. Braun S. (2020). Analysing interactional phenomena in video remote interpreting in collaborative settings: implications for interpreter education. Interpreter Translator Trainer 14, 279–302. doi: 10.1080/1750399X.2020.1800364

[ref56] DeLeeuw K. E. Mayer R. E. (2008). A comparison of three measures of cognitive load: evidence for separable measures of intrinsic, extraneous, and germane load. J. Educ. Psychol. 100, 223–234. doi: 10.1037/0022-0663.100.1.223

[ref57] Desmet B. Vandierendonck M. Defrancq B. (2018). “Simultaneous interpretation of numbers and the impact of technological support,” in Interpreting and Technology. ed. Fantinuoli C. (Language Science Press), 13–27.

[ref58] Díaz-Galaz S. (2011). The effect of previous preparation in simultaneous interpreting: preliminary results. Across Lang. Cult. 12, 173–191. doi: 10.1556/Acr.12.2011.2.3

[ref59] Díaz-Galaz S. Padilla P. Bajo M. T. (2015). The role of advance preparation in simultaneous interpreting: a comparison of professional interpreters and interpreting students. Interpreting 17, 1–25. doi: 10.1075/intp.17.1.01dia

[ref60] Dong Y. Liu Y. Cai R. (2018). How does consecutive interpreting training influence working memory: a longitudinal study of potential links between the two. Front. Psychol. 9:875. doi: 10.3389/fpsyg.2018.00875 29922199 PMC5996275

[ref61] Dragsted B. Hansen I. (2009). Exploring translation and interpreting hybrids. The case of sight translation. Meta 54, 588–604. doi: 10.7202/038317ar

[ref62] Ehrensberger-Dow M. Albl-Mikasa M. Andermatt K. Heeb A. H. Lehr C. (2020). Cognitive load in processing ELF: translators, interpreters, and other multilinguals. J. English Lingua Franca 9, 217–238. doi: 10.1515/jelf-2020-2039

[ref63] Elmer S. Klein C. Kühnis J. Liem F. Meyer M. Jäncke L. (2014). Music and language expertise influence the categorization of speech and musical sounds: behavioral and electrophysiological measurements. J. Cogn. Neurosci. 26, 2356–2369. doi: 10.1162/jocn_a_00632, PMID: 24702451

[ref64] Ericsson K. A. Simon H. A. (1998). How to study thinking in everyday life: contrasting think-aloud protocols with descriptions and explanations of thinking. Mind Cult. Act. 5, 178–186. doi: 10.1207/s15327884mca0503_3

[ref65] Fantinuoli C. (2018). “Computer-assisted interpreting: challenges and future perspectives,” in Trends in E-tools and Resources for Translators and Interpreters. eds. Corpas Pastor G. Durán-Muñoz I. (Leiden: Brill), 153–174.

[ref66] Firth A. (1996). The discursive accomplishment of normality: on ‘lingua franca’ English and conversation analysis. J. Pragmat. 26, 237–259. doi: 10.1016/0378-2166(96)00014-8

[ref67] Flavell J. H. (1976). “Metacognitive aspects of problem solving,” in The Nature of Intelligence. ed. Resnick L. (Mahwah, NJ: Lawrence Erlbaum Associates), 231–236.

[ref68] Fujita R. (2021). The role of speech-in-noise in Japanese EFL learners’ listening comprehension process and their use of contextual information. Int. J. Listening 36, 118–137. doi: 10.1080/10904018.2021.1963252

[ref69] Gany F. Kapelusznik L. Prakash K. Gonzalez J. Orta L. Y. Tseng C. H.. (2007). The impact of medical interpretation method on time and errors. J. Gen. Intern. Med. 22, 319–323. doi: 10.1007/s11606-007-0361-7, PMID: 17957418 PMC2078536

[ref70] Gerver D. (1975). A psychological approach to simultaneous interpretation. Meta 20, 119–128. doi: 10.7202/002885ar

[ref71] Gerver D. (1976). “Empirical studies of simultaneous interpretation: a review and a model,” in Translation: Applications and Research. ed. Brislin R. W. (New York, NY: Gardiner), 165–207.

[ref72] Gilbert J. K. (2004). Models and modelling: routes to more authentic science education. Int. J. Sci. Math. Educ. 2, 115–130. doi: 10.1007/s10763-004-3186-4

[ref73] Gile D. (1995). Basic Concepts and Models for Interpreter and Translator Training. Amsterdam: John Benjamins Publishing.

[ref74] Gile D. (1999). Testing the effort models’ tightrope hypothesis in simultaneous interpreting – a contribution. Hermes 23, 153–172.

[ref75] Gile D. (2008). Local cognitive load in simultaneous interpreting and its implications for empirical research. Forum 6, 59–77. doi: 10.1075/forum.6.2.04gil

[ref76] Gile D. (2009). Basic Concepts and Models for Interpreter and Translator Training. Amsterdam: John Benjamins Publishing.

[ref77] Gluszek A. Dovidio J. F. (2010). The way they speak: a social psychological perspective on the stigma of nonnative accents in communication. Personal. Soc. Psychol. Rev. 14, 214–237. doi: 10.1177/1088868309359288, PMID: 20220208

[ref78] Gracia-García R. A. (2002). “Telephone interpreting: a review of pros and cons,” in Proceedings of the 43rd Annual Conference. ed. Scott B. (Alexandria: American Translators Association), 195–216.

[ref79] Hart S. G. Staveland L. E. (1988). “Development of NASA-TLX (task load index): results of empirical and theoretical research,” in Human mental Workload. eds. Hancock P. A. Meshkati N. (Amsterdam: North-Holland), 139–183.

[ref80] Havelka I. (2020). Video-mediated remote interpreting in healthcare: analysis of an Austrian pilot project. Babel 66, 326–345. doi: 10.1075/babel.00156.hav

[ref81] Hervais-Adelman A. Moser-Mercer B. Golestani N. (2018). Commentary: Broca pars triangularis constitutes a “hub” of the language-control network during simultaneous language translation. Front. Hum. Neurosci. 12:22. doi: 10.3389/fnhum.2018.00022, PMID: 29441007 PMC5797666

[ref83] Huddle A. P. White M. D. Rogers F. (2000). Using a teaching model to correct known misconceptions in electrochemistry. J. Chem. Educ. 77, 104–110. doi: 10.1021/ed077p104

[ref84] Hymes D. (1972). “On communicative competence,” in Sociolinguistics. eds. Pride J. Holmes J. (Harmondsworth: Penguin Books), 269–293.

[ref85] Hymes D. (1974). Foundations in Sociolinguistics: An Ethnographic Approach. Philadelphia, PA: University of Pennsylvania Press.

[ref86] Hyönä J. Tommola J. Alaja A.-M. (1995). Pupil dilation as a measure of processing load in simultaneous interpretation and other language tasks. Q. J. Exp. Psychol. 48, 598–612. doi: 10.1080/14640749508401407, PMID: 7568993

[ref87] Ivanova A. (2000). “The use of retrospection in research on simultaneous interpreting,” in Tapping and Mapping the Processes of Translation and Interpreting: Outlooks. eds. Tirkkonen-Condit S. Jääskeläinen R. (Benjamins Translation Library), 27–52.

[ref88] Jenkins J. (2000). The Phonology of English as an International Language. Oxford: Oxford University Press.

[ref89] Jiménez-Ivars A. (2021). Telephone interpreting for asylum seekers in the US: a corpus-based study. J. Specialised Transl. 36a, 125–146.

[ref90] Jones D. Gill P. Harrison R. Meakin R. Wallace P. (2003). An exploratory study of language interpretation services provided by videoconferencing. J. Telemed. Telecare 9, 51–56. doi: 10.1258/135763303321159701 12641894

[ref91] Kahneman D. (1973). Attention and Effort Prentice-Hall.

[ref92] Kalina S. (2000). Interpreting competences as a basis and a goal for teaching. Interpreters’ Newsletter 14, 3–32.

[ref93] Kalina S. (2002). “Quality in interpreting and its prerequisites a framework for a comprehensive view,” in Interpreting in the 21st Century: Challenges and Opportunities. eds. Garzone G. Viezzi M. (Amsterdam: John Benjamins), 121–130.

[ref94] Kalina S. (2005). Quality assurance for interpreting processes. Meta 50, 768–784. doi: 10.7202/011017ar

[ref95] Kalyuga S. Plass J. L. (2018). “Cognitive load as a local characteristic of cognitive processes: implications for measurement approaches” in Cognitive load Measurement and Application: A Theoretical Framework for Meaningful Research and Practice. ed. Zheng R. Z. (New York, NY: Routledge), 59–74.

[ref96] Ketola A. (2015). Translation diaries of an illustrated technical text: translation students’ conceptions of word-image interaction. Connexions: Int. Prof. Commun. J. 3, 13–40.

[ref97] Kirchhoff H. (1976). “Simultaneous interpreting: interdependence of variables in the interpreting process, interpreting models and interpreting strategies,” in The Interpreting Studies reader. eds. Pöchhacker F. Schlesinger M. (London: Routledge), 111–119.

[ref98] Ko L. (2006). The need for long-term empirical studies in remote interpreting research: a case study of telephone interpreting. Linguistica Antverpiensia, New Series–Themes in Translation Studies 5, 325–338. doi: 10.52034/lanstts.v5i.167

[ref100] Korpal P. (2016). Interpreting as a stressful activity: physiological measures of stress in simultaneous interpreting. Poznan Stud. Contemp. Ling. 52, 297–316. doi: 10.1515/psicl-2016-0011

[ref101] Korpal P. Jasielska A. (2019). Investigating interpreters’ empathy: are emotions in simultaneous interpreting contagious? Targets 31, 2–24. doi: 10.1075/target.17123.kor

[ref004] Korpal P. Stachowiak-Szymczak K. (2018). The whole picture: Processing of numbers and their context in simultaneous interpreting. Pozn. Stud. Contemp. Linguist. 54, 335–354. doi: 10.1515/psicl-2018-0013

[ref005] Korpal P. Stachowiak-Szymczak K. (2019). Combined problem triggers in simultaneous interpreting: Exploring the effect of delivery rate on processing and rendering numbers. Perspect. 28, 126–143. doi: 10.1080/0907676X.2019.1628285

[ref102] Korpal P. Stachowiak-Szymczak K. (2020). Combined problem triggers in simultaneous interpreting: exploring the effect of delivery rate on processing and rendering numbers. Perspect. 28, 126–143. doi: 10.1080/0907676X.2019.1628285

[ref103] Koskinen K. Ruokonen M. (2017). “Love letters or hate mail? Translators’ technology acceptance in the light of their emotional narratives” in Human Issues in Translation Technology. ed. Kenny D. (London: Routledge), 26–42.

[ref104] Krell M. Xu K. M. Rey G. D. Paas F. (2022). “Editorial: recent approaches for assessing cognitive load from a validity perspective” in Recent Approaches for Assessing Cognitive load from a Validity Perspective. eds. Krell M. Xu K. M. Rey G. D. Paas F. (Lausanne, Frontiers Media SA), 4–10.

[ref105] Kuang H. Zheng B. (2022). Note-taking effort in video remote interpreting: effects of source speech difficulty and interpreter work experience. Perspectives, 1–21. doi: 10.1080/0907676X.2022.2053730

[ref106] Kurz I. (1994). “A look into the “black box”- EEC probability mapping during mental simultaneous interpreting” in Translation Studies – An Interdiscipline. eds. Snell-Hornby M. Pochhacker M. Kaindl K. (Amsterdam: John Benjamins), 199–213.

[ref107] Kurz I. (1995). Watching the brain at work-an exploratory study of EEG changes during simultaneous interpreting (SI). Interpreters’ Newsletter 6, 3–16.

[ref108] Lamberger-Felber H. (2001). Text-oriented research into interpreting-examples from a case-study. HERMES-J. Lang. Commun. Bus. 14, 39–64. doi: 10.7146/hjlcb.v14i26.25638

[ref109] Lee J. Aryadoust V. Ng L. Y. Foo S. (2020). A neurocognitive comparison of listening to academic lectures and natural sounds: implications for test validity. Int. J. Listening, 1–15. doi: 10.1080/10904018.2020.1818565

[ref110] Leppink J. Paas F. van der Vleuten C. P. M. van Gog T. van Merriënboer J. J. G. (2013). Development of an instrument for measuring different types of cognitive load. Behav. Res. Methods 45, 1058–1072. doi: 10.3758/s13428-013-0334-1, PMID: 23572251

[ref111] Leung C. (2005). Language and content in bilingual education. Linguist. Educ. 16, 238–252. doi: 10.1016/j.linged.2006.01.004

[ref112] Li X. (2018). Teaching beliefs and learning beliefs in translator and interpreter education: an exploratory case study. Interpreter Translator Trainer 12, 132–151. doi: 10.1080/1750399X.2017.1359764

[ref113] Licoppe C. Veyrier C. A. (2017). How to show the interpreter on screen? The normative organization of visual ecologies in multilingual courtrooms with video links. J. Pragmat. 107, 147–164. doi: 10.1016/j.pragma.2016.09.012

[ref114] Lin Y. Lv Q. Liang J. (2018). Predicting fluency with language proficiency, working memory, and directionality in simultaneous interpreting. Front. Psychol. 9:1543. doi: 10.3389/fpsyg.2018.01543, PMID: 30186213 PMC6110880

[ref115] Linton P. M. Plamondon B. D. Dick A. O. Bittner A. C. Christ R. E. (1989). “Operator workload for military system acquisition,” in Applications of Human Performance Models to System design. eds. McMillan G. R. Beevis D. Breda L. Van, Cham: Springer.

[ref116] Locatis C. Williamson D. Gould-Kabler C. Zone-Smith L. Detzler I. Roberson J.. (2010). Comparing in-person, video, and telephonic medical interpretation. J. Gen. Intern. Med. 25, 345–350. doi: 10.1007/s11606-009-1236-x, PMID: 20107916 PMC2842540

[ref117] Low A. R. L. Aryadoust V. (2021). Investigating test-taking strategies in listening assessment: a comparative study of eye-tracking and self-report questionnaires. Int. J. Listening, 1–20. doi: 10.1080/10904018.2021.1883433

[ref118] Luximon A. Goonetilleke R. S. (2001). Simplified subjective workload assessment technique. Ergonomics 44, 229–243. doi: 10.1080/00140130010000901, PMID: 11219757

[ref119] Ma X. Li D. (2021). A cognitive investigation of ‘chunking’and ‘reordering’for coping with word-order asymmetry in English-to-Chinese sight translation: evidence from an eye-tracking study. Interpreting 23, 192–221. doi: 10.1075/intp.00057.ma

[ref120] Ma X. Li D. Hsu Y. Y. (2021). Exploring the impact of word order asymmetry on cognitive load during Chinese–English sight translation: evidence from eye-movement data. Targets 33, 103–131. doi: 10.1075/target.19052.ma

[ref121] Martin S. (2018). “A critical analysis of the theoretical construction and empirical measurement of cognitive load,” in Cognitive load Measurement and Application: A Theoretical Framework for Meaningful Research and Practice. ed. Zheng R. Z. (London: Routledge), 29–44.

[ref122] Mazza C. (2001). Numbers in simultaneous interpretation. Interpreters’ Newsletter 14, 87–104.

[ref123] McNamara D. S. de Vega M. O’Reilly T. (2007). “Comprehension skill, inference making, and the role of knowledge,” in Higher level Language Processes in the brain: Inference and Comprehension Processes. eds. Schmalhofer F. Perfetti C. A. (Mahwah, NJ: Erlbaum), 233–253.

[ref006] Mellinger C. D. Hanson T. A. (2018). Interpreter traits and the relationship with technology and visibility. Transl. Interpret. Stud. 13, 366–392. doi: 10.1075/tis.00021.mel

[ref124] Mellinger C. D. Hanson T. A. (2019). Meta-analyses of simultaneous interpreting and working memory. Interpreting 21, 165–195. doi: 10.1075/intp.00026.mel

[ref125] Meshkati N. (1988). “Toward development of a cohesive model of workload,” in Human Mental Workload. eds. Hancock P. A. Meshkati N. (Amsterdam: North–Holland), 305–314.

[ref126] Meuleman C. Van Besien F. (2009). Coping with extreme speech conditions in simultaneous interpreting. Interpreting 11, 20–34. doi: 10.1075/intp.11.1.03meu

[ref127] Mizuno A. (2005). Process model for simultaneous interpreting and working memory. Meta 50, 739–752. doi: 10.7202/011015ar

[ref007] Moray N. (1967). Where is capacity limited: A survey and a model. Acta Psychol. 27, 84–92. 10.1016/0001-6918(67)90048-0 6062244

[ref128] Moser B. (1978). “Simultaneous Interpretation: a hypothetical model and its practical application,” in Language Interpretation and Communication. Proceedings of the NATO Symposium. eds. Gerver D. Sinaiko H. W. (New York, NY: Plenum Press), 353–368.

[ref129] Moser-Mercer B. (1997). “Skill components in simultaneous interpreting,” in Conference Interpreting: Current Trends in Research. eds. Gambier Y. Gile D. Taylor C. (Amsterdam: John Benjamins), 133–148.

[ref130] Moser-Mercer B. (2000). Simultaneous interpreting: cognitive potential and limitations. Interpreting 5, 83–94. doi: 10.1075/intp.5.2.03mos

[ref131] Moser-Mercer B. (2003). Remote interpreting: Assessment of human factors and performance parameters. Available at: https://aiic.net/page/1125/remote-interpreting-assessment-of-human-factors-and-pe/lang/1 (Accessed July 6, 2021).

[ref132] Moser-Mercer B. (2005a). Remote interpreting: the crucial role of presence. Bull. VALS-ASLA 81, 73–97.

[ref133] Moser-Mercer B. (2005b). Remote interpreting: issues of multi-sensory integration in a multilingual task. Meta 50, 727–738. doi: 10.7202/011014ar

[ref134] Moser-Mercer B. Frauenfelder U. H. Casado B. Kunzli A. (2000). “Searching to define expertise in interpreting,” in Language Processing and Simultaneous Interpreting. eds. Englund Dimitrova B. Hyltenstam K. (Amsterdam: John Benjamins), 107–131.

[ref135] Mouzourakis P. (1996). Videoconferencing: techniques and challenges. Interpreting 1, 21–38. doi: 10.1075/intp.1.1.03mou

[ref136] Mouzourakis P. (2003). That feeling of being there: vision and presence in remote interpreting. Available at: http://www.aiic.net/ViewPage.cfm?page_id=1173 (Accessed July 6, 2021).

[ref137] Mouzourakis P. (2006). Remote interpreting: a technical perspective on recent experiments. Interpreting 8, 45–66. doi: 10.1075/intp.8.1.04mou

[ref138] Muñoz E. Calvo N. García A. M. (2018). Grounding translation and interpreting in the brain: what has been, can be, and must be done. Perspectives 27, 483–509. doi: 10.1080/0907676X.2018.1549575

[ref0013] Napier J. (2002). University interpreting: Linguistic issues for consideration, The Journal of Deaf Studies and Deaf Education. The Journal of Deaf Studies and Deaf Education 7, 281–301. doi: 10.1093/deafed/7.4.281 15451866

[ref139] Napier J. Skinner R. Braun S. (Eds.). (2018). Here or There: Research on Interpreting Via Video Link. Washington, DC: Gallaudet University Press.

[ref140] Oléron P. Nanpon H. (1965). Recherches sur la traduction simultanée [studies of simultaneous translation]. J. Psychol. Norm. Pathol. 62, 73–94. 14302791

[ref141] Ouwehand K. van der Kroef A. Wong J. Paas F. (2022). Measuring cognitive load: are there more valid alternatives to Likert rating scales? Front. Psychol. 6, 146–158. doi: 10.3389/feduc.2021.702616

[ref142] Oviatt S. L. Cohen P. R. (1992). Spoken language in interpreted telephone dialogues. Comput. Speech Lang. 6, 277–302. doi: 10.1016/0885-2308(92)90021-U

[ref143] Ozolins U. (2011). Telephone interpreting: understanding practice and identifying research needs. Transl. Interpreting 3, 33–47.

[ref144] Paas F. Tuovinen J. E. Tabbers H. Van Gerven P. W. (2003). Cognitive load measurement as a means to advance cognitive load theory. Educ. Psychol. 38, 63–71. doi: 10.1207/S15326985EP3801_8

[ref145] Paas F. G. W. C. Van Merriënboer J. J. G. (1994). Instructional control of cognitive load in the training of complex cognitive tasks. Educ. Psychol. Rev. 6, 351–371. doi: 10.1007/BF02213420

[ref146] Paas F. van Merriënboer J. J. (2020). Cognitive-load theory: methods to manage working memory load in the learning of complex tasks. Curr. Dir. Psychol. Sci. 29, 394–398. doi: 10.1177/0963721420922183

[ref147] Parsons K. McCormac A. Butavicius M. Pattinson M. Jerram C. (2014). Determining employee awareness using the human aspects of information security questionnaire (HAIS-Q). Comput. Secur. 42, 165–176. doi: 10.1016/j.cose.2013.12.003

[ref148] Pio S. (2003). The relation between ST delivery rate and quality in simultaneous interpretation. Interpreters’ Newsl. 14, 69–100.

[ref149] Plevoets K. Defrancq B. (2018). The cognitive load of interpreters in the European Parliament: a corpus-based study of predictors for the disfluency uh (m). Interpreting 20, 1–32. doi: 10.1075/intp.00001.ple

[ref150] Pöchhacker F. (2016). Introducing Interpreting Studies. 2nd Edn. London: Routledge.

[ref151] Powell M. B. Manger B. Dion J. Sharman S. J. (2017). Professionals’ perspectives about the challenges of using interpreters in child sexual abuse interviews. Psychiatry Psychol. Law 24, 90–101. doi: 10.1080/13218719.2016.1197815 PMC6818322 31983941

[ref152] Prandi B. (2018). “An exploratory study on CAI tools in simultaneous interpreting: theoretical framework and stimulus validation,” in Interpreting and Technology. ed. Fantinuoli C. (Berlin: Language Science Press), 29–59.

[ref153] Pretorius A. Cilliers P. J. (2007). Development of a mental workload index: a systems approach. Ergonomics 50, 1503–1515. doi: 10.1080/00140130701379055, PMID: 17654038

[ref154] Price C. J. Green D. W. von Studnitz R. (1999). A functional imaging study of translation and language switching. Brain 122, 2221–2235. doi: 10.1093/brain/122.12.2221, PMID: 10581218

[ref155] Reid G. B. Nygren T. E. (1988). “The subjective workload assessment technique: a scaling procedure for measuring mental workload,” in Human Mental Workload. eds. Hancock P. A. Meshkati N. (Amsterdam: North-Holland), 185–218.

[ref156] Riccardi A. (1998). “Interpreting strategies and creativity,” in Translators’ Strategies and Creativity: Selected Papers from the 9th International Conference on Translation and Interpreting. eds. Beylard-Ozeroff A. Králová J. Moser-Mercer B. (Amsterdam: Benjamins Translation Library), 171–180.

[ref157] Riccardi A. (2005). On the evolution of interpreting strategies in simultaneous interpreting. Meta 50, 753–767. doi: 10.7202/011016ar

[ref158] Rinne J. O. Tommola J. Laine M. Krause B. J. Schmidt D. Kaasineni V.. (2000). The translating brain: cerebral activation patterns during simultaneous interpreting. Neurosci. Lett. 294, 85–88. doi: 10.1016/S0304-3940(00)01540-8, PMID: 11058793

[ref159] Rojo López A. M. Foulquié-Rubio A. I. Espín López L. Martínez Sánchez F. (2021). Analysis of speech rhythm and heart rate as indicators of stress on student interpreters. Perspectives 29, 591–607. doi: 10.1080/0907676X.2021.1900305

[ref160] Rojo López A. M. Martín R. M. (2022). “Translation process research,” in The Routledge Handbook of Translation and Methodology. eds. Zanettin F. Rundle C. (London: Routledge), 356–372.

[ref161] Rosiers A. Eyckmans J. Bauwens D. (2011). A story of attitudes and aptitudes? Investigating individual difference variables within the context of interpreting. Interpreting 13, 53–69. doi: 10.1075/intp.13.1.04ros

[ref162] Roziner I. Shlesinger M. (2010). Much ado about something remote: stress and performance in remote interpreting. Interpreting 12, 214–247. doi: 10.1075/intp.12.2.05roz

[ref163] Runcieman A. J. (2020). Community Interpreting and the Covid-19 crisis: Present relevancy and future directions. Tilburg Papers in Culture Studies, 242, 1–22. https://pure.uvt.nl/ws/portalfiles/portal/48995997/TPCS_242_Runcieman.pdf

[ref164] Schnaubert L. Schneider S. (2022). Analysing the relationship between mental load or mental effort and metacomprehension under different conditions of multimedia design. Front. Psychol. 6, 159–177. doi: 10.3389/feduc.2021.648319

[ref166] Schultheis H. Jameson A. (2004). “Assessing cognitive load in adaptive hypermedia systems: physiological and behavioral methods,” in Adaptive Hypermedia and Adaptive web-based Systems. eds. Bra P. D. E. De Nejdl W. (Berlin: Springer) 225–234.

[ref008] Seeber K. (2017). “Multimodal processing in simultaneous interpreting,” in The handbook of translation and cognition. eds J. W. Schwieter and A. Ferreira (Wiley Blackwell), 84–92.

[ref167] Seeber K. G. (2011). Cognitive load in simultaneous interpreting: existing theories – new models. Interpreting. 13, 176–204. doi: 10.1075/intp.13.2.02see

[ref168] Seeber K. G. (2013). Cognitive load in simultaneous interpreting: measures and methods. Targets 25, 18–32. doi: 10.1075/target.25.1.03see

[ref169] Seeber K. G. Keller L. Amos R. Hengl S. (2019). Expectations vs. experience: attitudes towards video remote conference interpreting. Interpreting 21, 270–304. doi: 10.1075/intp.00030.see

[ref170] Seeber K. G. Keller L. Hervais-Adelman A. (2020). When the ear leads the eye–the use of text during simultaneous interpretation. Lang. Cognit. Neurosci. 35, 1480–1494. doi: 10.1080/23273798.2020.1799045

[ref171] Seeber K. G. Kerzel D. (2011). Cognitive load in simultaneous interpreting: model meets data. Int. J. Biling. 16, 228–242. doi: 10.1177/1367006911402982

[ref172] Seleskovitch D. Lederer M. (1989). Pdagogie raisonne de l’interprtation [Reasoned Pedagogy of Interpretation]. Paris: Didier rudition.

[ref173] Setton R. (1999). Simultaneous Interpretation: A Cognitive-pragmatic Analysis. Amsterdam: Benjamins Publishing.

[ref174] Setton R. (2001). Deconstructing SI: a contribution to the debate on component processes. Interpreters’ Newsl. 11, 1–26.

[ref175] Setton R. (2002). Pragmatic analysis as a methodology: a reply to Gile’s review of Setton (1999). Targets 14, 353–360. doi: 10.1075/target.14.2.08set

[ref176] Shlesinger M. (2000). “Interpreting as a cognitive process: how can we know what really happens?” in Tapping and Mapping the Processes of Translation and Interpreting: Outlooks. eds. Tirkkonen-Condit S. Jääskeläinen R. (Amsterdam: Benjamins Translation library), 3–16.

[ref177] Skinner R. Napier J. Braun S. (2018). “Interpreting via video link: mapping of the field,” in Here or there: Research on Interpreting via Video Link. eds. Napier J. Skinner R. Braun S. (Washington DC: Gallaudet University Press), 11–35.

[ref178] Su W. Li D. (2019). Identifying translation problems in English-Chinese sight translation: an eye-tracking experiment. Translation and interpreting studies. J. Am. Transl. Interpreting Stud. Assoc. 14, 110–134. doi: 10.1075/tis.00033.su

[ref179] Sweller J. (1988). Cognitive load during problem solving: effects on learning. Cogn. Sci. 12, 257–285. doi: 10.1016/0364-0213(88)90023-7

[ref180] Sweller J. (2018). “The role of independent measures of load in cognitive load theory,” in Cognitive Load Measurement and Application: A Theoretical Framework for Meaningful Research and Practice. ed. Zheng R. Z. (London: Routledge), 3–8.

[ref181] Sweller J. Ayres P. Kalyuga S. (2011). Cognitive Load Theory. Berlin: Springer.

[ref182] Sweller J. van Merrienboer J. Paas F. (1998). Cognitive architecture and instructional design. Educ. Psychol. Rev. 10, 251–296. doi: 10.1023/A:1022193728205

[ref183] Sweller J. van Merriënboer J. J. Paas F. (2019). Cognitive architecture and instructional design: 20 years later. Educ. Psychol. Rev. 31, 261–292. doi: 10.1007/s10648-019-09465-5

[ref184] Timarová S. (2008). “Working memory and simultaneous interpreting,” in Translation and its Others: Selected Papers of the CETRA Research Seminar in Translation Studies. ed. Boulogne P. (Belgium: KU Leuven Centre for Translation Studies), 1–28.

[ref185] Timarová S. Salaets H. (2011). Learning styles, motivation and cognitive flexibility in interpreter training: self-selection and aptitude. Interpreting 13, 31–52. doi: 10.1075/intp.13.1.03tim

[ref186] Tiselius E. Sneed K. (2020). Gaze and eye movement in dialogue interpreting: an eye-tracking study. Biling. Lang. Congn. 23, 780–787. doi: 10.1017/S1366728920000309

[ref187] Tymoczko M. (2012). The neuroscience of translation. Target 24, 83–102. doi: 10.1075/target.24.1.06tym

[ref188] Van de Putte E. De Baene W. García-Pentón L. Woumans E. Dijkgraaf A. Duyck W. (2018). Anatomical and functional changes in the brain after simultaneous interpreting training: a longitudinal study. Cortex 99, 243–257. doi: 10.1016/j.cortex.2017.11.024, PMID: 29291529

[ref189] Van Merriënboer J. J. Kester L. Paas F. (2006). Teaching complex rather than simple tasks: balancing intrinsic and germane load to enhance transfer of learning. Appl. Cogn. Psychol. 20, 343–352. doi: 10.1002/acp.1250

[ref190] Vianna B. (2005). Simultaneous interpreting: A relevance-theoretic approach. Intercult. Pragmat. 2, 169–190. doi: 10.1515/iprg.2005.2.2.169

[ref191] Vranjes J. Brône G. Feyaerts K. (2018a). Dual feedback in interpreter-mediated interactions: on the role of gaze in the production of listener responses. J. Pragmat. 134, 15–30. doi: 10.1016/j.pragma.2018.06.002

[ref192] Vranjes J. Brône G. Feyaerts K. (2018b). On the role of gaze in the organization of turn-taking and sequence organization in interpreter-mediated dialogue. Lang. Dialogue 8, 439–467. doi: 10.1075/ld.00025.vra

[ref193] Vranjes J. Oben B. (2022). Anticipation and timing of turn-taking in dialogue interpreting: a quantitative study using mobile eye-tracking data. Targets. doi: 10.1075/target.20121.vra

[ref194] Wadensjö C. (1999). Telephone interpreting & the synchronization of talk in social interaction. Translator 5, 247–264. doi: 10.1080/13556509.1999.10799043

[ref009] Wang J. (2018). “It keeps me on my toes”: Interpreters’ perceptions of challenges in telephone interpreting and their coping strategies. Target 30, 430–462. doi: 10.1075/target.17012.wan

[ref195] Wang J. Antonenko P. Keil A. Dawson K. (2020). Converging subjective and psychophysiological measures of cognitive load to study the effects of instructor-present video. Mind Brain Educ. 14, 279–291. doi: 10.1111/mbe.12239

[ref196] Wen H. Dong Y. (2019). How does interpreting experience enhance working memory and short-term memory: a meta-analysis. J. Cogn. Psychol. 31, 769–784. doi: 10.1080/20445911.2019.1674857

[ref0010] Weng Y. Zheng B. Dong Y. (2022). Time pressure in translation: Psychological and physiological measures. Target. doi: 10.1075/target.20148.wen

[ref197] Wessling D. M. Shaw S. (2014). Persistent emotional extremes and video relay service interpreters. J. Interpretation 23, 6.

[ref198] Wickens C. D. (1984). “Processing resources in attention” in Varieties of Attention. eds. Parasuraman R. Davies D. R. (New York: Academic Press), 63–102.

[ref199] Wickens C. D. (2002). Multiple resources and performance prediction. Theor. Issues Ergon. Sci. 3, 159–177. doi: 10.1080/14639220210123806

[ref200] Xie B. Salvendy G. (2000). Review and reappraisal of modelling and predicting mental workload in single-and multi-task environments. Work Stress 14, 74–99. doi: 10.1080/026783700417249

[ref201] Yin B. Chen F. Ruiz N. Ambikairajah E. (2008). “Speech-based cognitive load monitoring system,” in 2008 IEEE International Conference on Acoustics, Speech and Signal Processing (Piscataway, NJ: IEEE), 2041–2044.

[ref202] Young M. S. Brookhuis K. A. Wickens C. D. Hancock P. A. (2015). State of science: mental workload in ergonomics. Ergonomics 58, 1–17. doi: 10.1080/00140139.2014.956151, PMID: 25442818

[ref203] Young M. S. Stanton N. A. (2005). “Mental workload,” in Handbook of human Factors and Ergonomics Methods. eds. Stanton N. A. Hedge A. Brookhuis K. Salas E. Hendrick H. W. (London: Taylor & Francis), 390–401.

[ref204] Zheng R. Z. (2018). Cognitive load Measurement and Application: A Theoretical Framework for Meaningful Research and Practice London, Routledge.

[ref0011] Zheng B. Zhou H. (2018). 隐喻表达过程：视译中听译时间差的眼动研究 [Revisiting processing time for metaphorical expressions: An eye-tracking study on eye-voice span during sight translation]. Wai Yu Jiao Xue Yu Yan Jiu [Foreign Language Teaching and Research] 50, 744–759. doi: 10.1016/j.bandc.2020.105584

[ref205] Zheng B. Báez S. Su L. Xiang X. Weis S. Ibáñez A.. (2020). Semantic and attentional networks in bilingual processing: fMRI connectivity signatures of translation directionality. Brain Cogn. 143:105584. doi: 10.1016/j.bandc.2020.105584 32485460 PMC7933822

[ref206] Zhu X. Aryadoust V. (In press) (2022). A Scientometric review of translation and interpreting research in the early 21st century. Targets.

